# Supplementary material for: Competitive binding and molecular crowding regulate the cytoplasmic interactome of non-viral polymeric gene delivery vectors
Source: Nat Commun. 2021 Nov 8;12:6445. doi: 10.1038/s41467-021-26695-w (PMC8576037; doi:10.1038/s41467-021-26695-w)
Supplement: Supplementary file 1 — Supplementary Information [file 41467_2021_26695_MOESM1_ESM.pdf]

Supplementary Information

Competitive Binding and Molecular Crowding Regulate the Cytoplasmic Interactome of Non-Viral Polymeric Gene Delivery Vectors

Aji Alex M. Raynold [1,2,#], *Danyang Li* [1,2,#], *Lan Chang* [1,2], *Julien E. Gautrot* [1,2*]

[1] Institute of Bioengineering and [2] School of Engineering and Materials Science, Queen Mary University of London, Mile End Road, London E1 4NS, United Kingdom

[#] These authors contributed equally to this work.

* To whom correspondence should be addressed. E-mail: j.gautrot@qmul.ac.uk

1. Supplementary Discussion

1.1 Interactome of PDMAEMA brush-functionalised nanoparticles and full cell lysates

As a comparison of the cytosolic interactome associated with polymer brush-functionalised nanoparticles, we also analysed the interactome of PDMAEMA brush-functionalised nanoparticles upon incubation in full cell lysates. We allowed PDMAEMA brush-functionalised nanoparticles to interact with full cell lysates (non-fractionated) prior to their separation via centrifugation and washing. Following desorption of the adsorbed molecules and digestion, protein analysis via mass spectrometry was performed and compared to the composition of pristine cell lysates (Supplementary Figures 2A and B). We identified 980 proteins present in the PDMAEMA brush-lysate proteome, 610 of which were detected with high Mascot scores in the initial lysate (in which we identified 2488 proteins). We selected the 63 proteins that were detected at high levels (> 10 fold that of the basal level within the sample, Supplementary Figure 2A). In agreement with results obtained with cytosolic fractions, 54 of these proteins are predicted to display a low isoelectric point (predicted average of 5.5), below 7.0 and the pKa of PDMAEMA brushes, therefore potentially adsorbing to PDMAEMA brushes via electrostatic interactions (Supplementary Figure 2B). However, 9 of the most abundant proteins in the vector proteome were predicted to display relatively high isoelectric points (as high as 12.1, Supplementary Figure 2B).

Similarly to results obtained for the proteome associated with polymer brush vectors from cytosolic fractions, analysis of the function of the highly abundant proteins identified in the vector interactome led us to group them into 3 main categories (Supplementary Figure 2A). The first category consisted in proteins involved in translation or directly binding RNA or DNA, including several members of the family of eukaryotic translation initiation factors and protein Niban 1. Two serine/arginine repetitive matrix proteins were also identified, with high predicted isoelectric points (11.8 and 12.1), and are essential to the function of pre- and post-splicing complexes[1]. Most of these proteins displayed relatively low abundance in the pristine lysate and were clearly enriched in the PDMAEMA/PMETAC brush-cytoplasmic proteome. Another group of proteins identified were associated with endosomes, the proteasome and lysosomes, or were localised in or at the Golgi and endoplasmic reticulum. These included the EH domain-binding protein 1, regulating endosomal trafficking[2], and the vacuolar protein sorting-associated protein 35, involved in protein sorting to avoid lysosomal degradation[3]. Some of these proteins, such as transmembrane protein 165, are membrane-associated proteins and may associate with the vectors during endocytosis or as they accumulate at the Golgi[4], directly or potentially through the binding of negatively charged phospholipid patches. Finally, a third group of proteins are cytoskeleton associated proteins, relatively acidic proteins with low predicted IPs, which may either associate via electrostatic interactions or

may associate to the endosome during transport towards the endoplasmic reticulum and nucleus. Interestingly, mitochondrial proteins were also found within the most abundant components of the vector-lysate interactome.

The distribution observed within the most abundant pool of proteins was also well reflected by cluster analysis (Supplementary Figure 3), with many of the proteins identified in the first two groups clustering together and highly enriched in the polycation proteome, compared to the pristine lysate. Further functional clustering analysis identified 43 gene groups (Supplementary Table 3) that could broadly be associated with the three main groups of proteins identified in Supplementary Figure 2A. In particular, most of the groups with high enrichment scores (above 20) were associated with translation, RNA/DNA binding or protein transport, endocytosis and exocytosis. Two groups with the lowest enrichment scores were associated with cell adhesion to the ECM (possibly internalised together with the vectors). In addition, analysis of the molecular weight distribution of the proteins identified within the PDMAEMA lysate proteome revealed a slight bias towards high molar masses compared to that of the pristine lysate (Supplementary Figure 2C). The peak of protein molecular weights occurred between 50 and 60 kDa for the PDMAEMA cytoplasmic adsorbate, compared to 20-30 kDa for the pristine lysate.

1.2 Stability of RNA adsorption as a function of pH and ionic strength

As previously observed, RNA adsorption was found to occur moderately fast to PDMAEMA brushes, as monitored by SPR (Figure 2D)[5]. Some desorption occurred in PBS, but lowering the pH to 5.5 did not result in any significant change in surface bound mass. This is in agreement with the pH stability of plasmid DNA complexes formed with PDMAEMA brushes[6]. This was confirmed by our fluorescence assay (Figure 2E). In contrast, raising the pH to 10.0 lead to the expected collapse of PDMAEMA brushes (Supplementary Figure 11) and the rapid release of RNA (Figure 2E), in agreement with neutralisation of this coating at pH above 9.0, their hydrophobic character and significant reduction in hydration[6]. Similarly, RNA binding was found to be stable at physiological ionic strength but oligonucleotides rapidly desorbed at high ionic strength (Figure 2F).

1.3 Model of oligonucleotide competitive desorption from polymer brushes

We revised a model of oligonucleotide adsorption to polymer brushes[5], taking into account the adsorption of molecules at charged interfaces[7], in which an equilibrium is established between molecules that have diffused to the subsurface and those adsorbed at the outer surface of the brush. Competitive adsorption occurs between molecules at the interface of the brushes and oligonucleotides and is regulated by diffusion between interfacial molecules and bulk solution molecules.

The kinetics model is outlined in Figure 3A.

The rate of adsorption/desorption of oligonucleotides at the surface is determined by:

$$\frac{d\Gamma}{dt} = k_a C_S[Br] - k_d C_{BO} - k_{cd} C_{BO}[A_{surf}] + k_{ca} C_S[A_{brush}] \qquad \text{(Supplementary Equation 1)}$$

Where Γ is the surface density of oligonucleotides, k_a and k_d are the surface adsorption and desorption rate constants, C_S is the bulk solution concentration of oligonucleotides (which is assumed to be equal to the subsurface concentration of oligonucleotides), C_{BO} is the surface concentration of oligonucleotides in the outer brush layer and $[Br]$ is the surface concentration of free (not occupied by oligonucleotides) brush sites.

The rate of desorption is derived from the following equations:

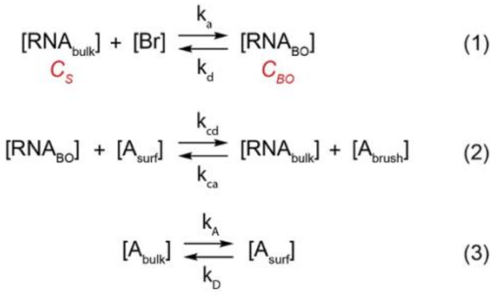

Where A is a competing molecule and $[A_{bulk}]$, $[A_{surf}]$ and $[A_{brush}]$ are the concentrations of competitor in the bulk, at the surface of the brush and in the brush, respectively.

In the steady-state, the flux of diffusion of oligonucleotides within the brush will be matched by the adsorption of new molecules at the brush outer surface:

$$k_{diff}(C_{BO} - C_{BI}) = k_a C_S[Br] - k_d C_{BO} \qquad \text{(Supplementary Equation 2)}$$

Where C_{BI} is the surface concentration of oligonucleotides at the inner brush layer and k_{diff} is the rate of diffusion of oligonucleotides within the brush.

Similarly, in the steady-state, the flux of diffusion of competing molecules A at the surface of the brush must be balanced by the rate of exchange with oligonucleotides in the brush.

$$k_A([A_{bulk}] - [A_{surf}]) = k_{cd} C_{BO}[A_{surf}] - k_{ca} C_S[A_{brush}] \qquad \text{(Supplementary Equation 3)}$$

It follows that the rate of desorption of oligonucleotides can be rewritten as (neglecting the surface concentration of competitor, compared to the large excess present in solution):

$$\frac{d\Gamma}{dt} = k_a C_S[Br] - k_d C_{BO} - k_A[A_{bulk}] \qquad \text{(Supplementary Equation 4)}$$

Considering that the initial surface concentration of free brush sites, $[Br]_0$, is the sum of $[Br]$ and Γ, and that Γ is the sum of the outer and inner brush layer oligonucleotide surface concentrations, C_{BO} and C_{BI}, this rearranges to give the following expression for C_{BO}:

$$C_{BO} = \frac{k_a C_S([Br]_0 - \Gamma) + k_{diff}\Gamma}{2\,k_{diff} + k_d} \qquad \text{(Supplementary Equation 5)}$$

Therefore, the rate of adsorption of oligonucleotides at the surface becomes:

$$\frac{d\Gamma}{dt} = k_a C_S([Br]_0 - \Gamma) - k_d \frac{k_a C_S([Br]_0 - \Gamma) + k_{diff}\Gamma}{2\, k_{diff} + k_d} - k_A[A_{bulk}]$$ (Supplementary Equation 6)

$$\frac{d\Gamma}{dt} = k_{app1} C_S [Br]_0 - (k_{app2} + k_{app1} C_S)\Gamma - k_A[A_{bulk}]$$ (Supplementary Equation 7)

Where k_{app1} and k_{app2} are two apparent rate constants:

$$k_{app1} = \frac{2\, k_a k_{diff}}{2\, k_{diff} + k_d}$$

$$k_{app2} = \frac{k_d k_{diff}}{2\, k_{diff} + k_d}$$

The initial surface concentration of free brush sites, $[Br]_0$, equivalent to the maximum number of oligonucleotides that can be bound in a brush, per surface area, can also be expressed as:

$$[Br]_0 = \sigma \frac{M_n^{Br}}{\alpha\, M_n^{ON}} = \frac{h\, \rho\, N_A}{\alpha\, M_n^{ON}}$$ (Supplementary Equation 8)

Where σ is the grafting density of the polymer brush, h its dry height, ρ its dry density and N_A the Avogadro number. The binding factor α defines the number of oligonucleotides of molecular weight M_n^{ON} that can be bound per polymer brush chain of molecular weight M_n^{Br}, as $\frac{M_n^{Br}}{\alpha\, M_n^{ON}}$.

Integration of the rate of desorption of oligonucleotides leads to the following expression for the evolution of the surface density of oligonucleotides as a function of time:

$$\Gamma = \frac{k_{app1} C_S [Br]_0 - k_A[A_{bulk}]}{k_{app3}} - \left(\frac{k_{app1} C_S [Br]_0 - k_A[A_{bulk}]}{k_{app3}} - \Gamma_{max}\right) e^{-k_{app3} t}$$ (Equation 1)

Where kapp1 and kapp2 are the two apparent rate constants and:

$$\Gamma_{max} = \frac{h\, \rho\, N_A\, k_{app1} C_S}{\alpha\, M_n^{ON}(k_{app2} + k_{app1} C_S)}$$ (Supplementary Equation 9)

$$k_{app3} = k_{app2} + k_{app1} C_S$$ (Supplementary Equation 10)

Γ_{max} had been derived from the adsorption kinetics, in the absence of competitors[5].

We can also derive the ultimate surface density of oligonucleotides Γ_∞ as:

$$\Gamma_\infty = \frac{k_{app1} C_S [Br]_0}{k_{app3}} - \frac{k_A}{k_{app3}} [A_{bulk}]$$ (Supplementary Equation 11)

This allows to derive k_A and k_{app3} from the evolution of Γ_∞ as a function of $[A_{bulk}]$.

2. Supplementary Figures

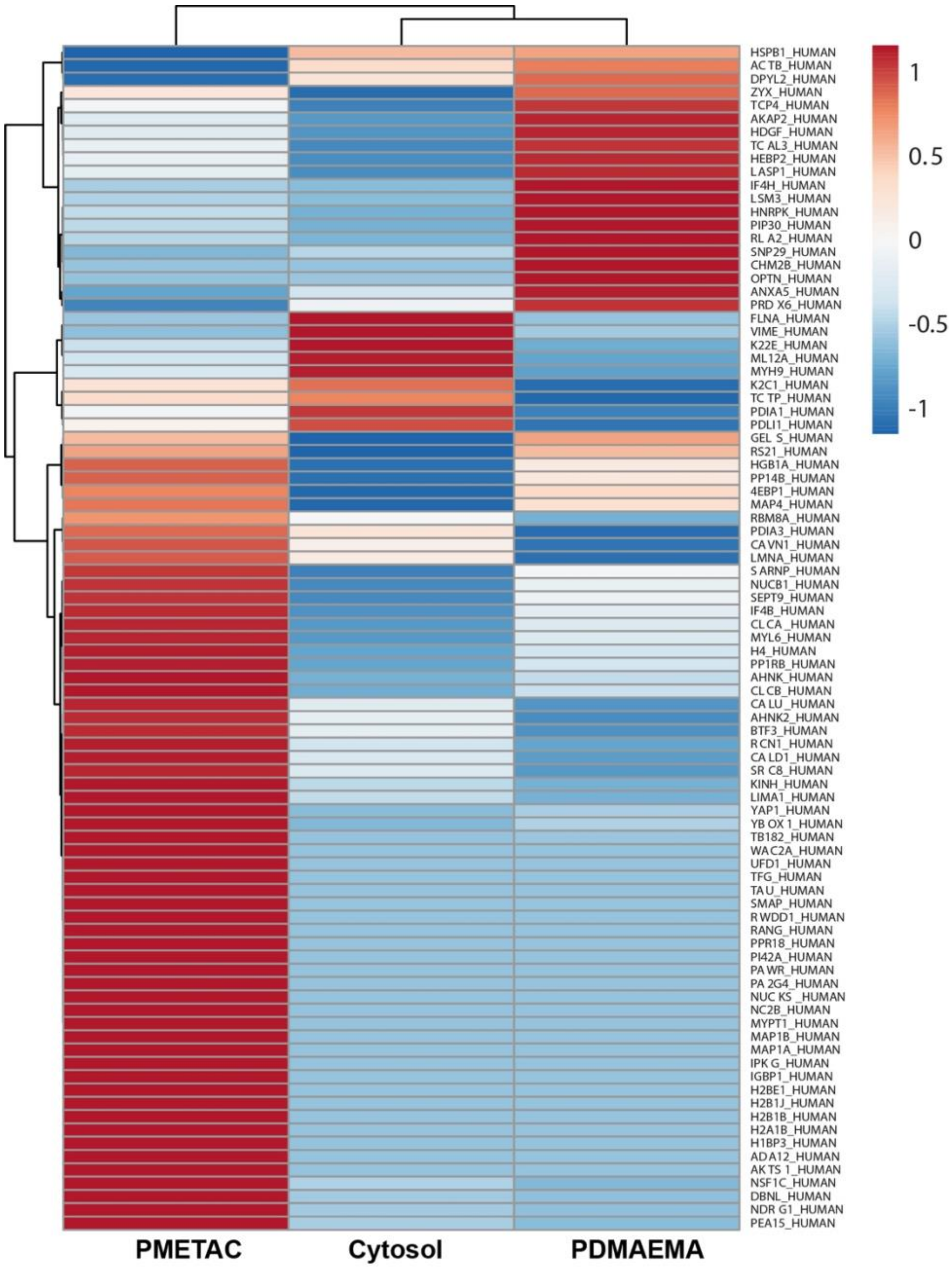

Supplementary Figure 1. Cluster analysis of the cytoplasmic proteome associated with PDMAEMA- and PMETAC-functionalised nanoparticles compared to that of pristine cytosolic fractions.

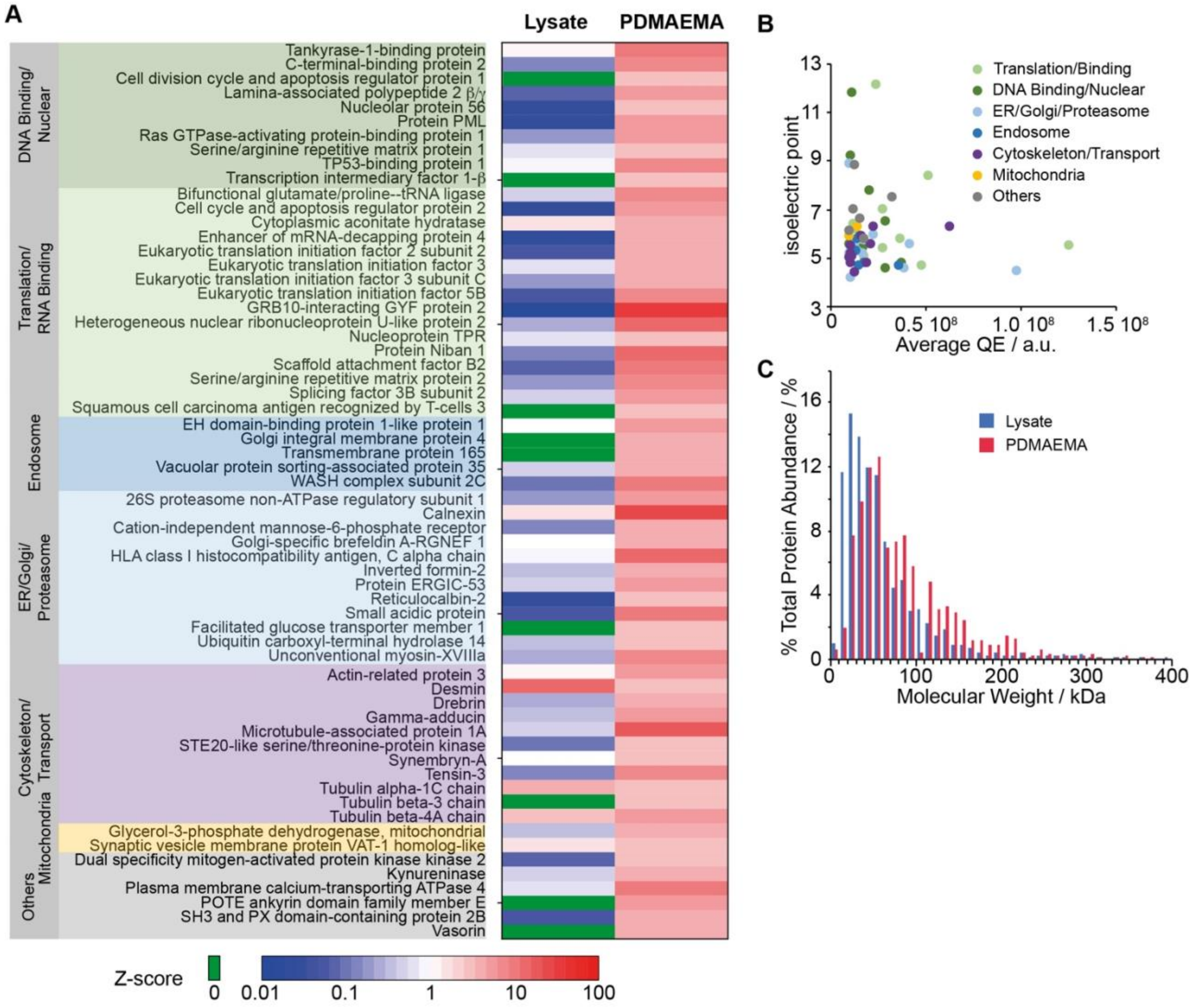

Supplementary Figure 2. Analysis of the polycationic vector interactome in full cell lysate. (A) Most abundant proteins identified by proteomics analysis of adsorbates to PDMAEMA-brush grafted nanoparticles, from cell lysates, compared to protein abundance in original lysates. (B) Correlation between the isoelectric point of the corresponding proteins and their abundance in the polycationic vector proteome. (C) Molecular weight distribution of proteins identified by proteomic analysis of cell lysates (blue) and PDMAEMA brush-adsorbate (red). Only proteins with a molecular weight below 400 kDa are reported.

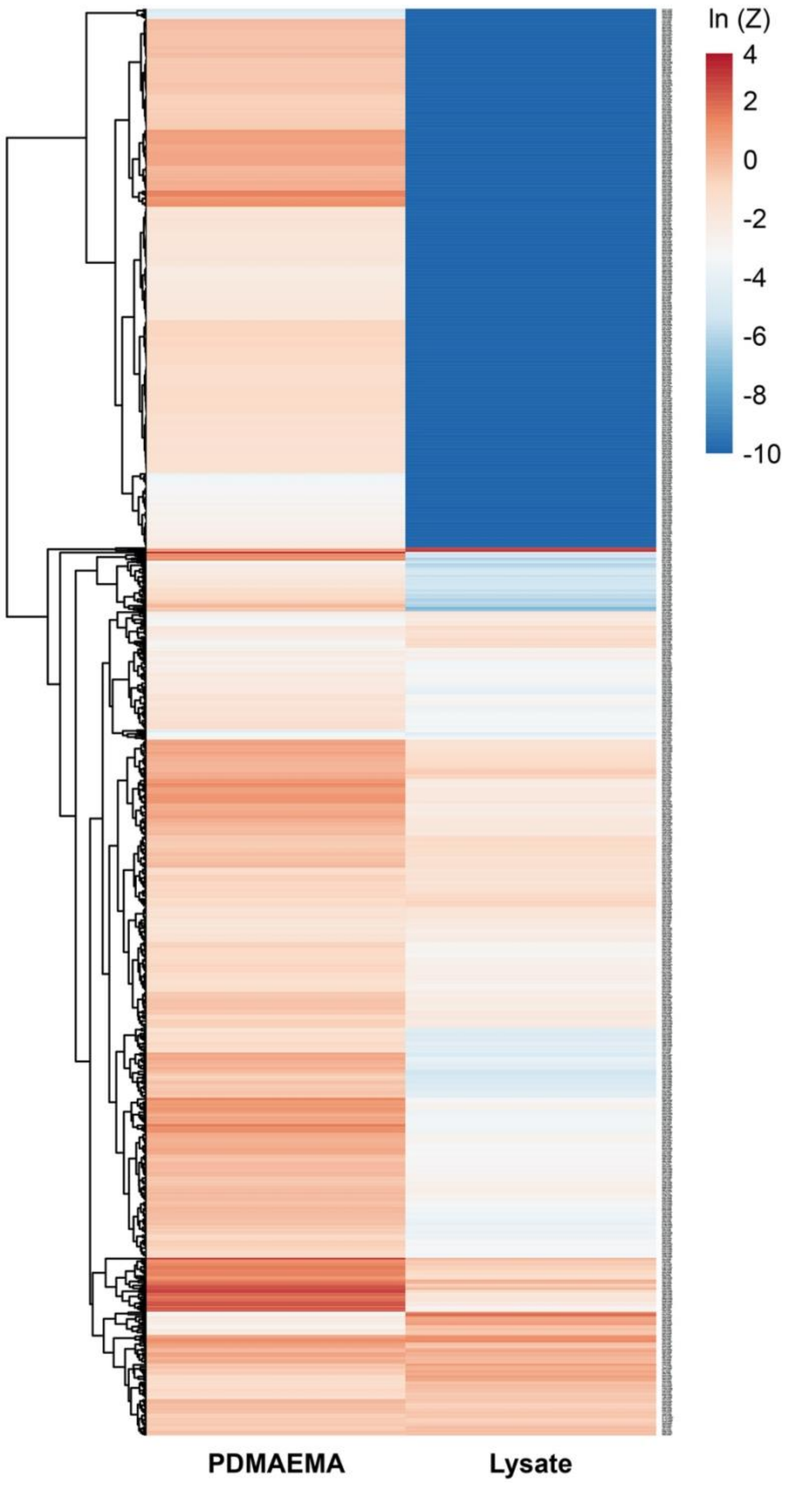

Supplementary Figure 3. Cluster analysis of the cell lysate proteome associated with PDMAEMA-functionalised nanoparticles compared to that of pristine lysates.

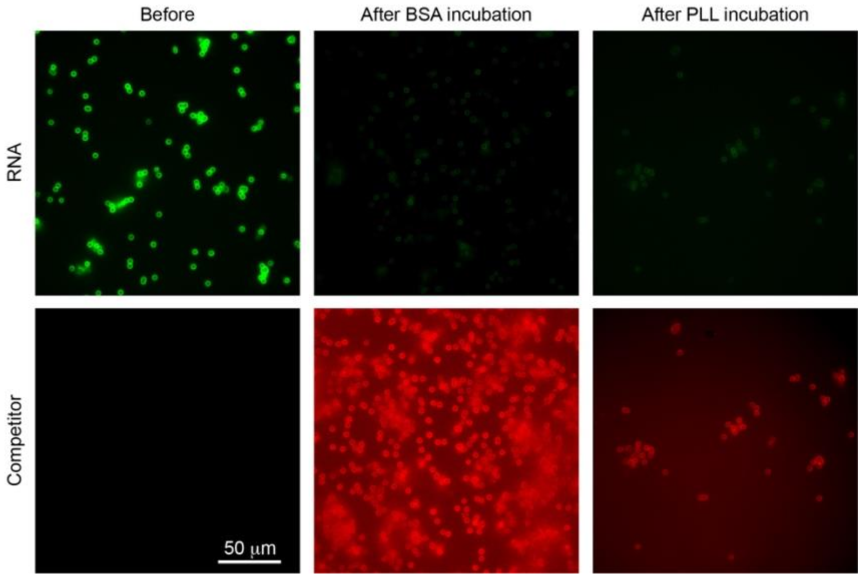

Supplementary Figure 4. Competitive adsorption regulate the composition of macromolecular corona surrounding polycationic brush delivery vectors and the displacement of oligonucleotides. RNA oligonucleotides (tagged with 6-FAM, green) were allowed to adsorb into the PDMAEMA brush-functionalised microparticles and samples were imaged ("Before"). Samples were then incubated into tagged BSA (Alexa Fluor™ 594, red) and PLL (Alexa Fluor™ 594, red) solutions. After removal of the macromolecule solutions and washing, samples were imaged again. Representative images from triplicate experiments.

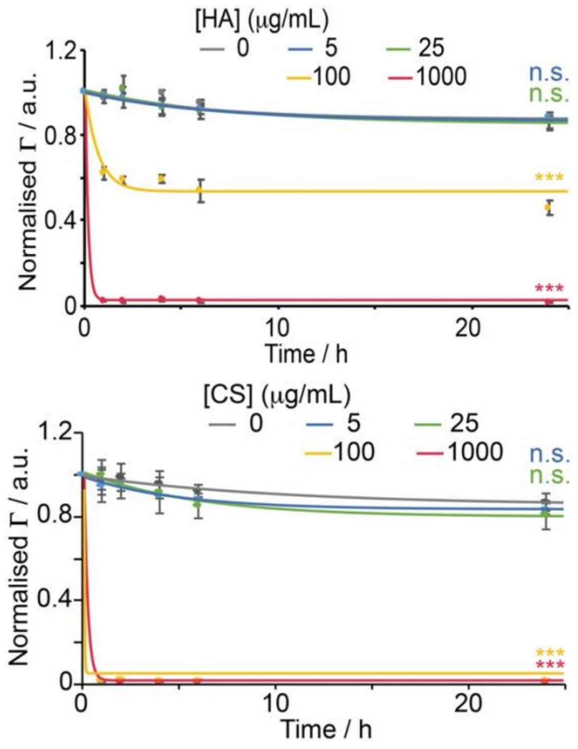

Supplementary Figure 5. Competitive displacement of RNA by hyaluronic acid (HA) and chondroitin sulfate (CS). Release profiles of cy-5 tagged siRNA from PDMAEMA brush grafted silica microparticles in the presence of hyaluronic acid (A) and chondroitin sulfate (B), as a function of competitor concentration. Lines shown are fit lines based on equation 1. Data are presented as mean values. Error bars are standard errors from triplicate experiments. n.s., not significant; ***, $p < 0.001$ (ANOVA); with respect to PBS alone.

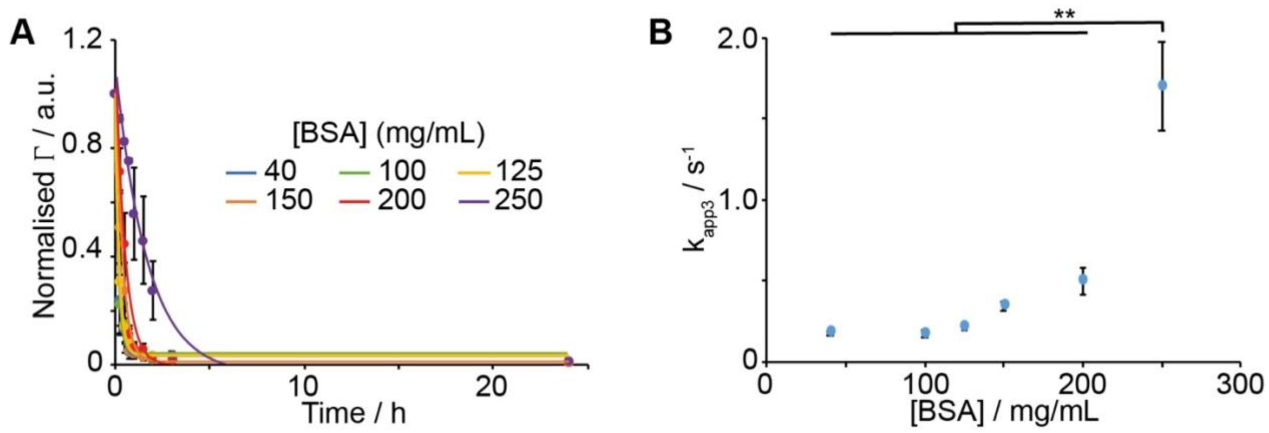

Supplementary Figure 6. Impact of molecular crowding on competitive binding. (A) Release profiles of cy5 siRNA from PDMAEMA brush-functionalised microparticles in the presence of heparin (20 μg/mL), at different concentrations of BSA. Lines are fits based on equation 1. B) Measured apparent rate constant (k_{app3}) extracted from fits, plotted as a function of [BSA]. Data are presented as mean values. Error bars are standard errors from triplicate experiments. **, $p < 0.01$ (ANOVA).

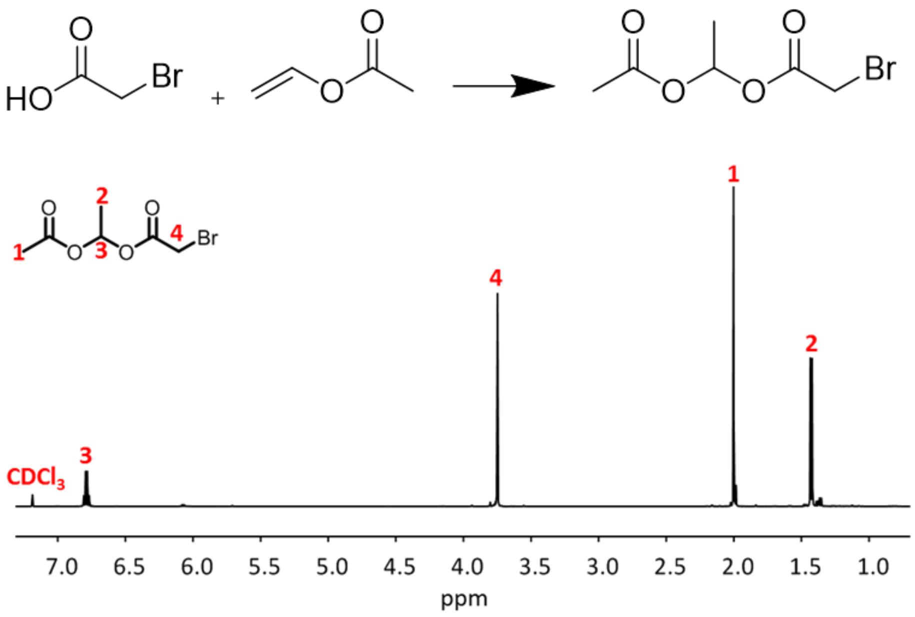

Supplementary Figure 7. Synthesis route and ^{1}H-NMR spectrum (in $CDCl_3$) of 1-acetoxyethyl-2-bromoacetate.

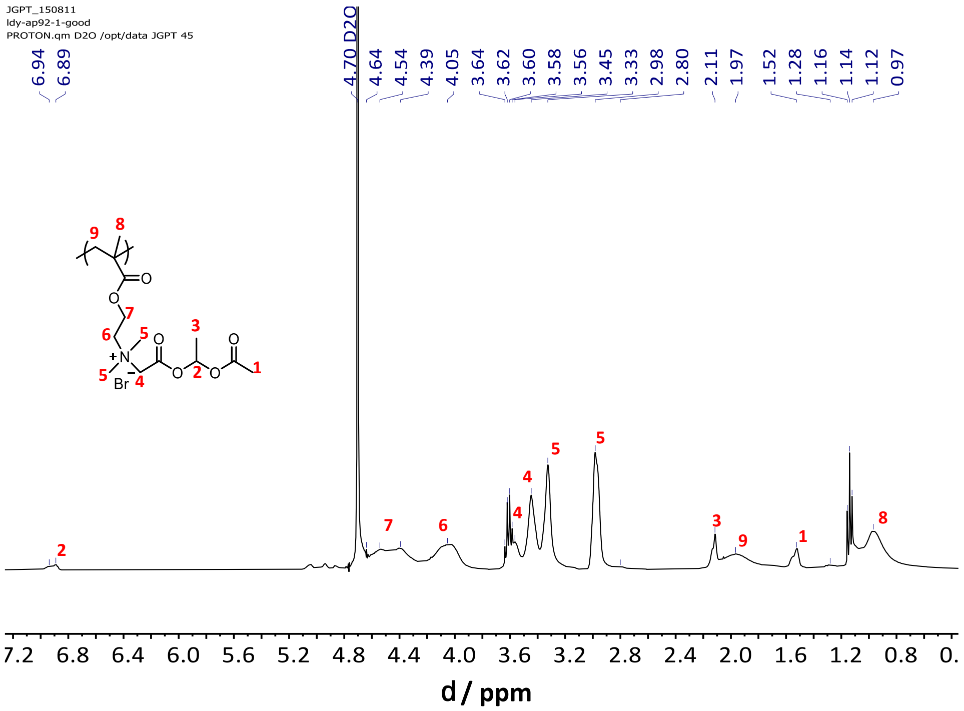

Supplementary Figure 8. ^{1}H-NMR spectrum of 1-acetoxyethyl-2-bromoacetate-functionalised free PDMAEMA (CS-PMETAC) in D_2O.

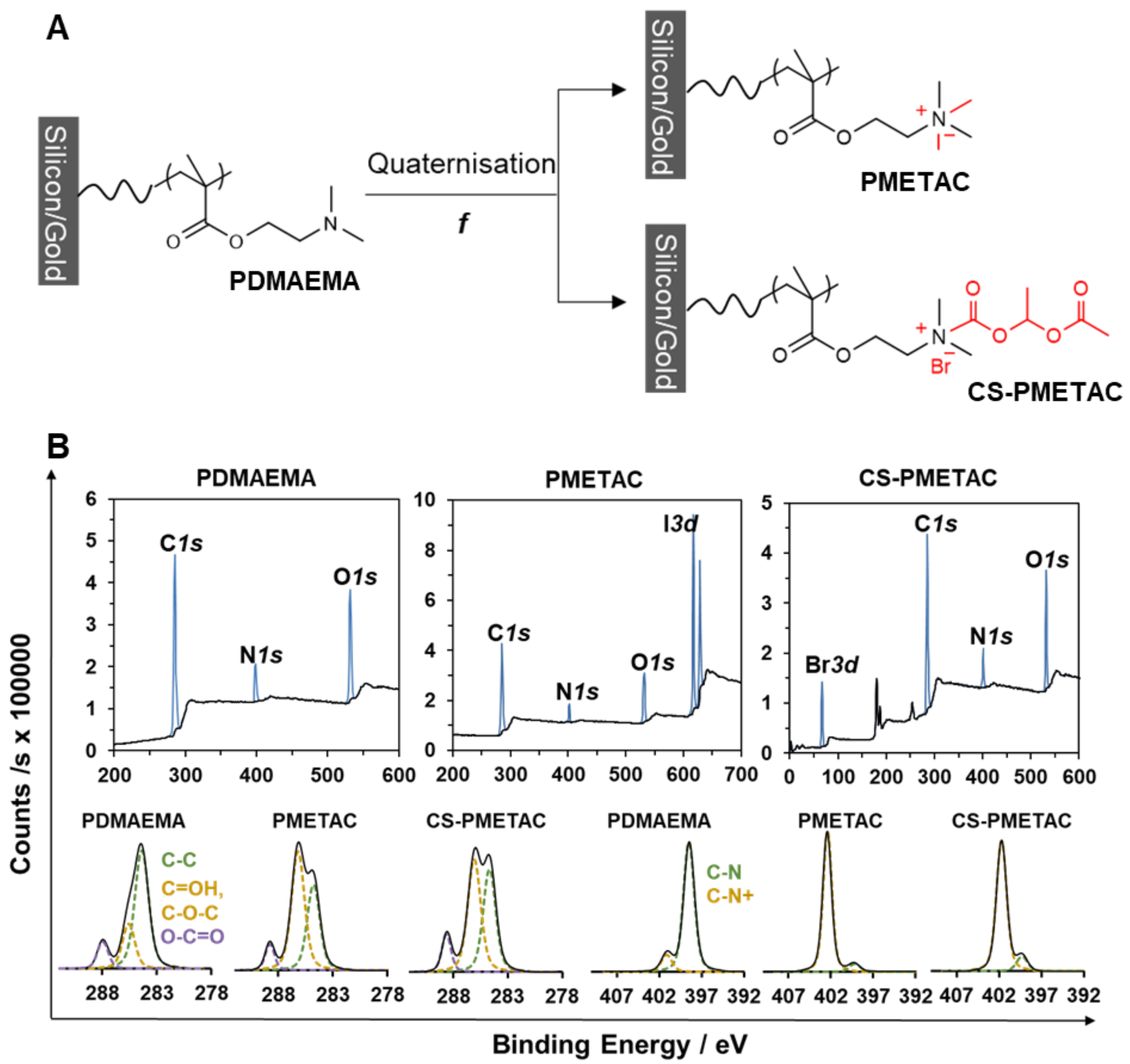

Supplementary Figure 9. (A) Formation of PMETAC and CS-PMETAC brushes from PDMAEMA; **(B)** Characterisation by XPS (wide scan spectrum, upper three figures; high-resolution spectra, lower six figures).

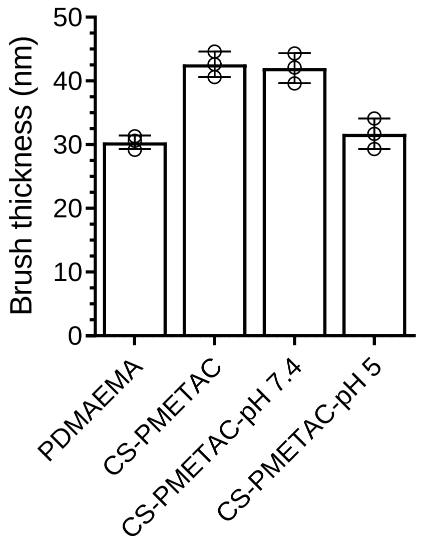

Supplementary Figure 10. pH responsiveness of CS-PMETAC brushes. Ellipsometric dry thickness of CS-PMETAC brushes grown from silicon substrates before and after 4 h incubation in buffer (PBS) with pH 5.0 or 7.4. Prior to measurements, chips were rinsed with DI water and dried in a N_2 stream. Data are presented as mean values. Error bars are standard errors from triplicate experiments.

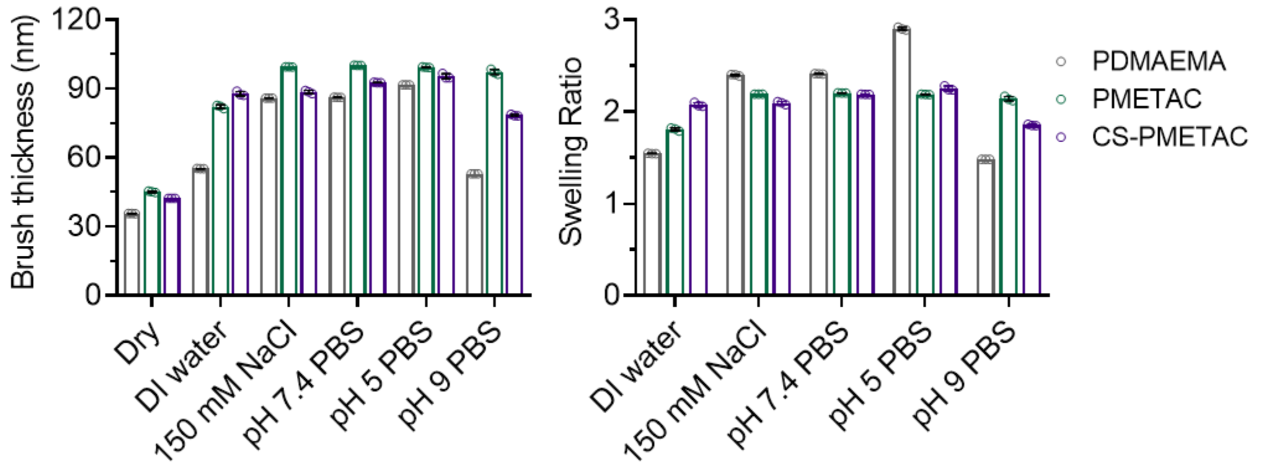

Supplementary Figure 11. (A) In situ ellipsometry measurements characterising the swelling of PDMAEMA, PMETAC and CS-PMETAC brushes in different buffers; **(B)** Corresponding swelling ratios calculated by comparing with dry brush thicknesses. Data are presented as mean values. Error bars are standard errors from triplicate experiments.

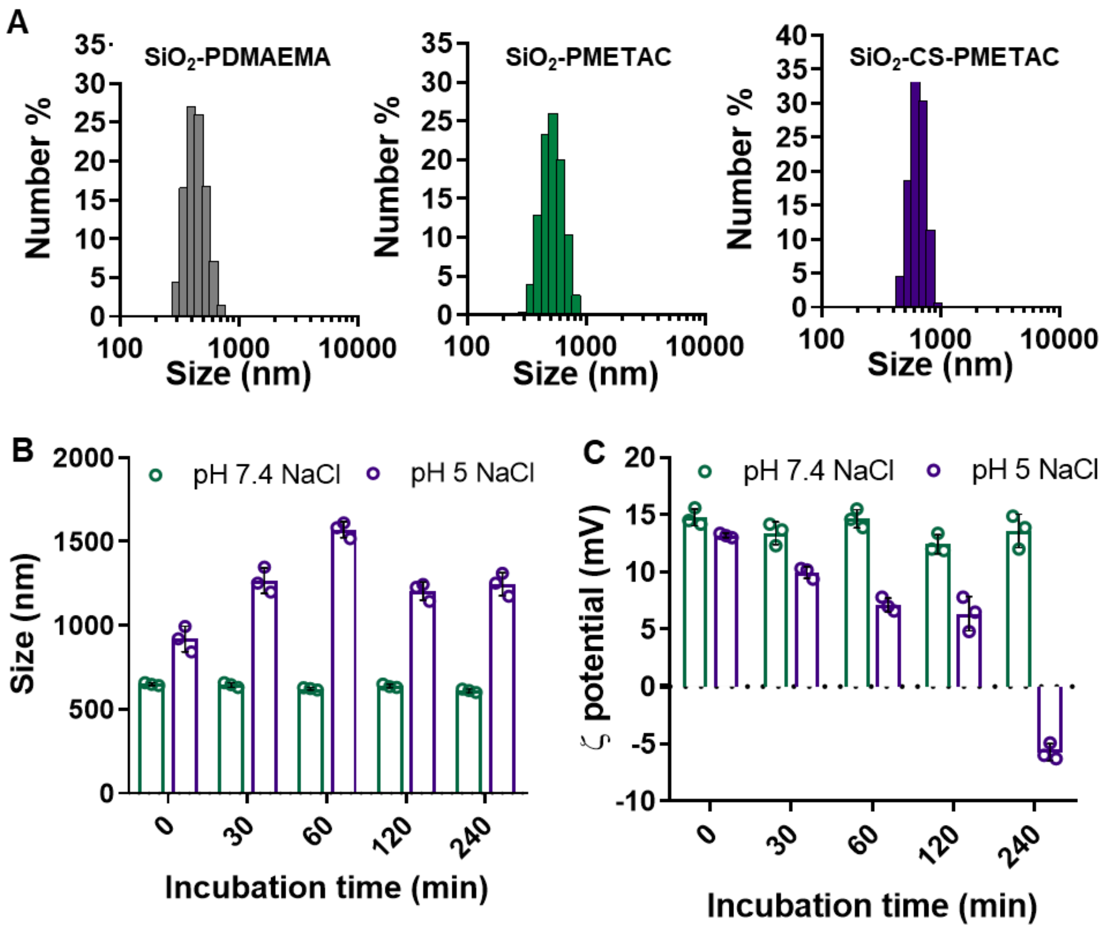

Supplementary Figure 12. (A) DLS measurements (number %) characterising the size distribution of PDMAEMA, PMETAC and CS-PMETAC brush-functionalised nanoparticles in PBS at 0.1 mg/mL at 25 °C; **(B)** and **(C)** Hydrodynamic diameters and *ζ*-potentials measured for CS-PMETAC brush-functinoalised nanoparticles incubated in pH 5.0 and 7.4 PBS buffers for 4 h. Data are presented as mean values. Error bars are standard errors from triplicate experiments.

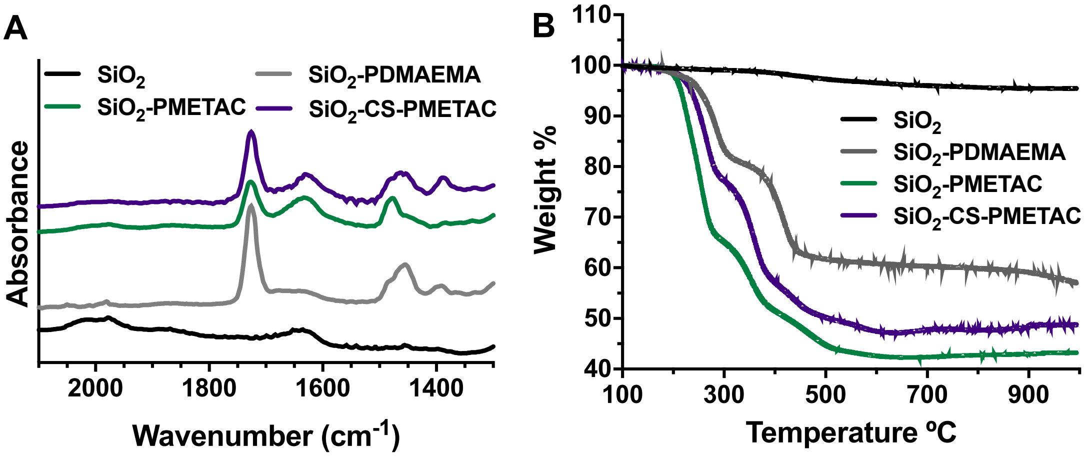

Supplementary Figure 13. (A) FTIR characterisation of bare, PDMAEMA, PMETAC and CS-PMETAC brush-functionalised nanoparticles in the range of 1300-2100 cm^{-1}; **(B)** TGA characterisation of bare, PDMAEMA, PMETAC and CS-PMETAC brush-functionalised nanoparticles recorded at a heating rate of 10 °C/min from room temperature to 1000 °C, weight loss was determined at 900 °C.

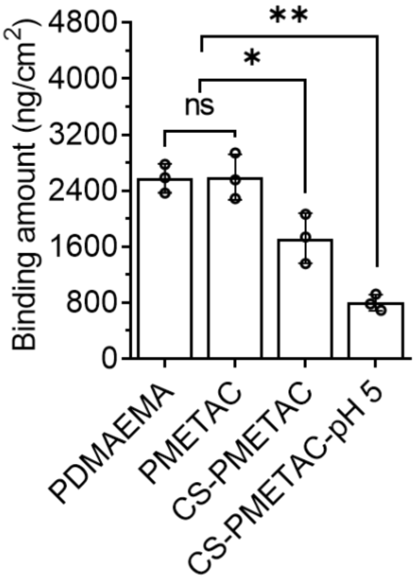

Supplementary Figure 14. Summary of surface coverage of siRNA bound to PDMAEMA, PMETAC and CS-PMETAC brushes in neutral PBS conditions and upon incubation in an acidic solution (pH 5.0), determine from SPR data. Data are presented as mean values. Error bars are standard errors from triplicate experiments. n.s., not significant; *, $p < 0.05$; **, $p < 0.01$ (ANOVA).

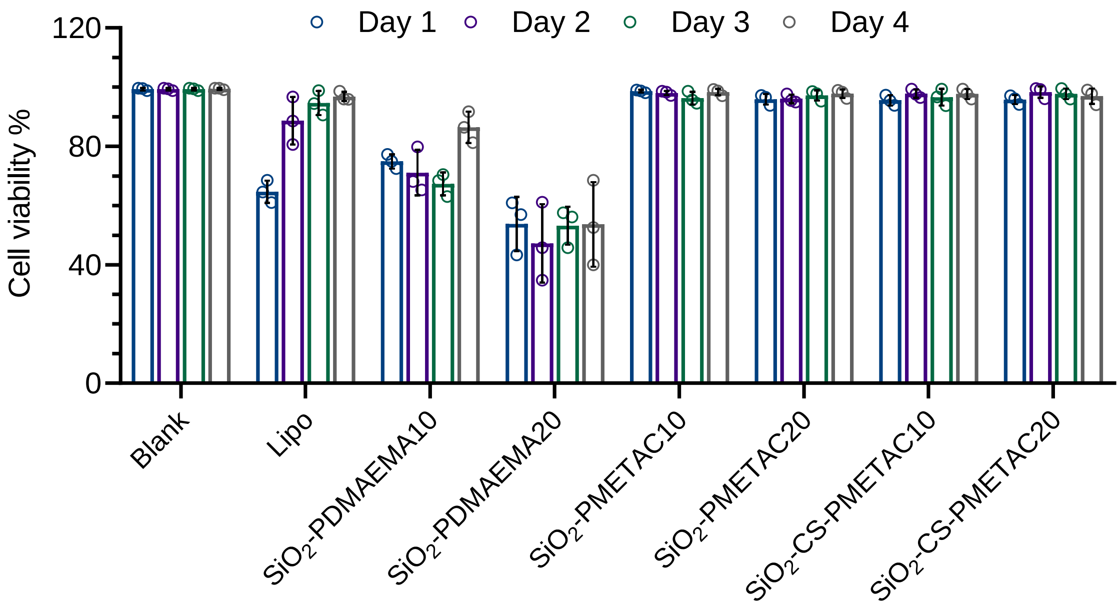

Supplementary Figure 15. HaCaT cell viability determined from live/dead assay. HaCaT cells were treated with PDMAEMA, PMETAC and CS-PMETAC brush-functionalised nanoparticles and lipofectamine, forming complexes with siRNA at N/P ratios of 10 and 20 and cultured from one to four days (n = 3, error bars are standard deviations). Data are presented as mean values. All cell viabilities were compared with the blank cell group.

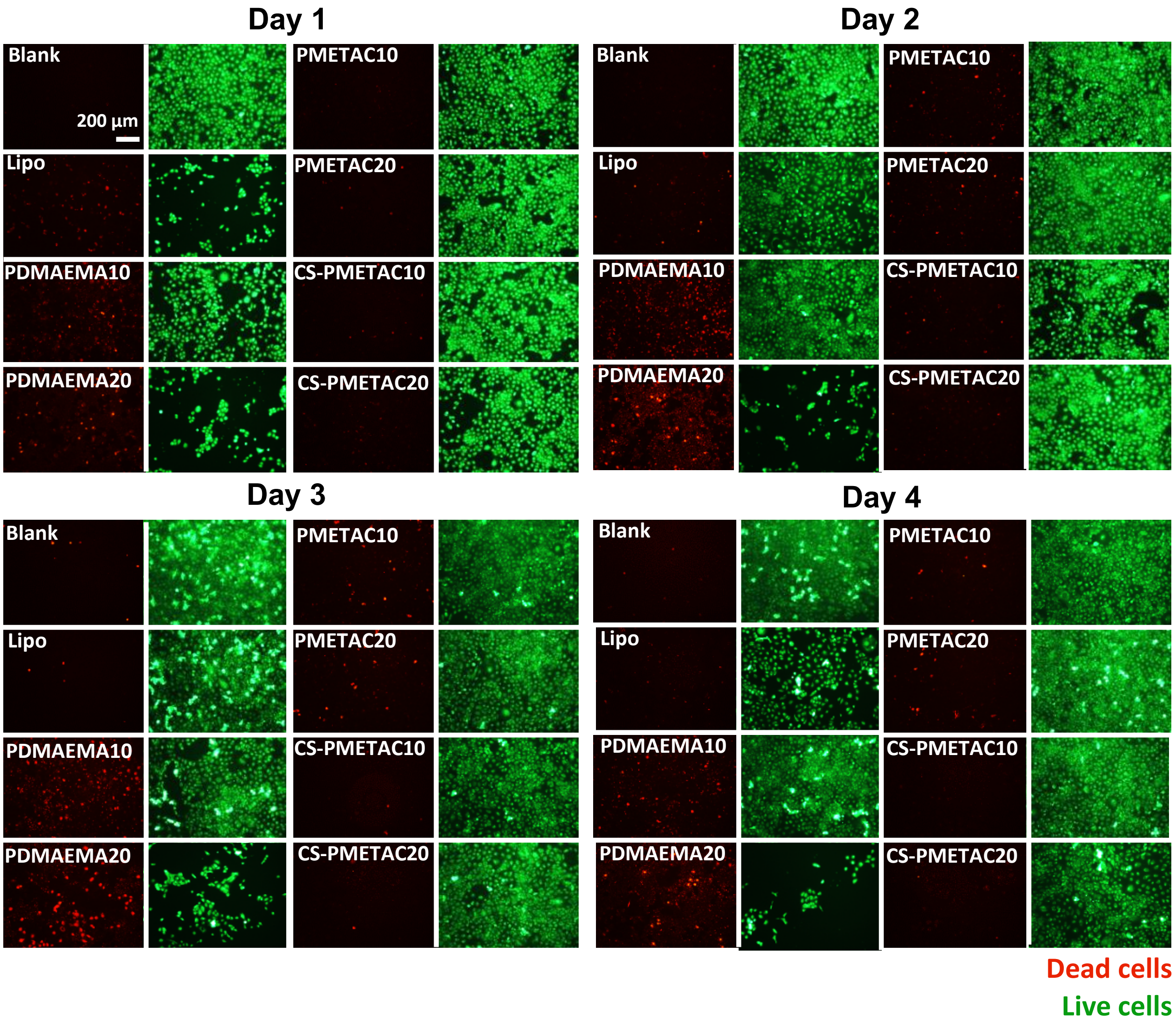

Supplementary Figure 16. Fluorescence images of HaCaT cells stained *via* live/dead assay (representative from triplicate experiments). HaCaT cells were treated with PDMAEMA, PMETAC and CS-PMETAC brush-functionalised nanoparticles and lipofectamine, forming complexes with siRNA at N/P ratios of 10 and 20 and cultured from one to four days. Scale bar: 200 µm.

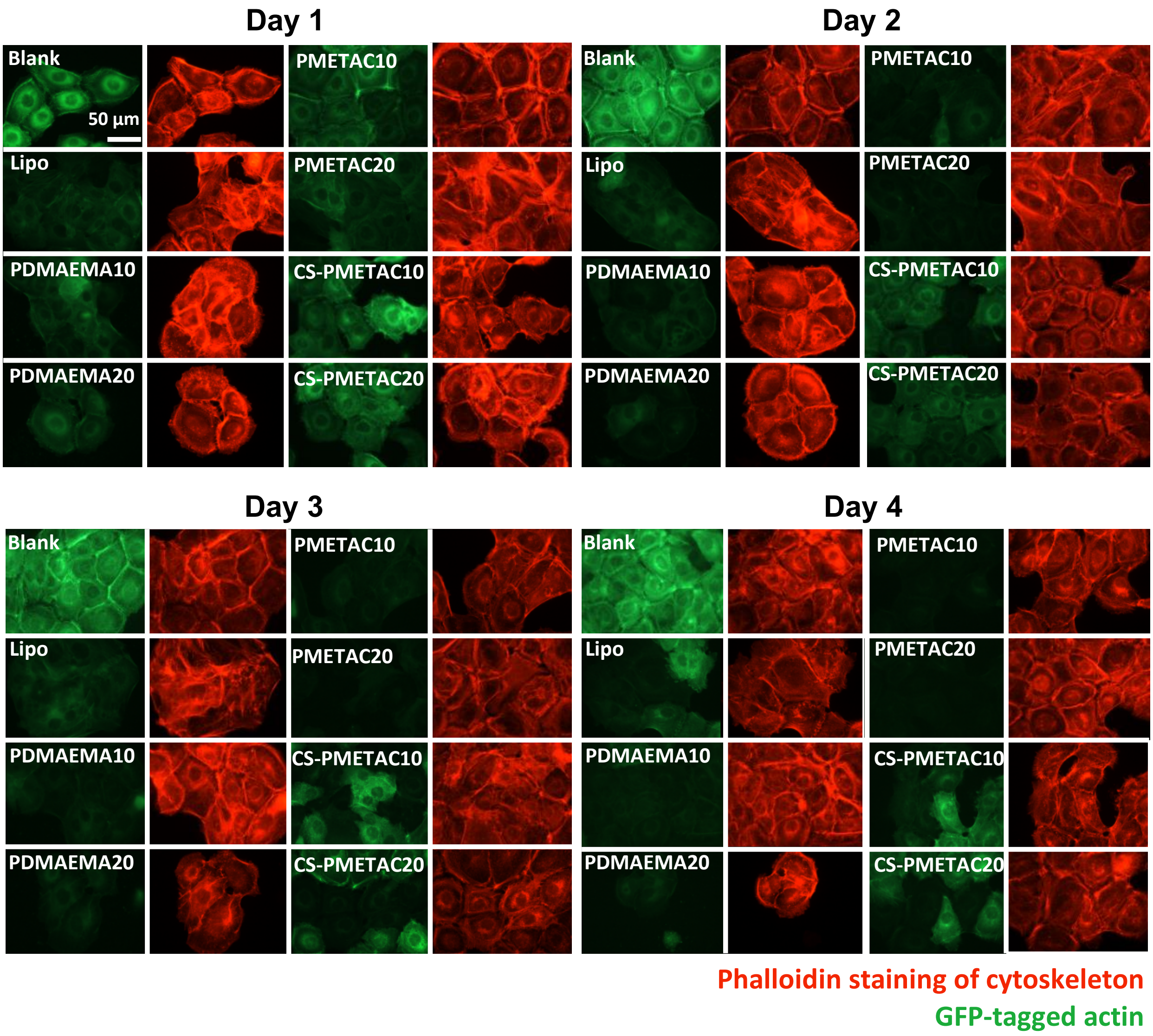

Supplementary Figure 17. Fluorescent images of HaCaT cells expressing GFP-actin and transfected with PDMAEMA, PMETAC and CS-PMETAC brush-functionalised nanoparticles and lipofectamine, forming complexes at N/P ratios of 10 and 20 (representative from triplicate experiments). Following transfection, cells were cultured for one to four days. Scale bar: 50 µm.

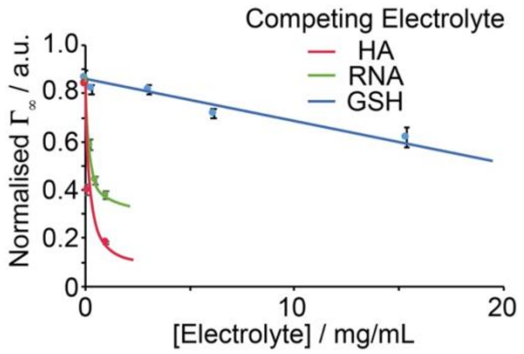

Supplementary Figure 18. Evolution of the residual surface density of oligonucleotide as a function of competitor concentration, plotted for HA, RNA and GSH. Data are presented as mean values. Error bars are standard errors from triplicate experiments.

Supplementary Table 1. Result of gene functional clustering of the cytoplasmic proteome. Colours indicate broad classes of functions associated with each cluster.

RNA/DNA binding / Translation

**Gene Group 9**	**Enrichment Score: 20.358704360292766**
SYYC_HUMAN	tyrosyl-tRNA synthetase(YARS)
SYAC_HUMAN	alanyl-tRNA synthetase(AARS)
SYNC_HUMAN	asparaginyl-tRNA synthetase(NARS)
SYTC_HUMAN	threonyl-tRNA synthetase(TARS)

**Gene Group 16**	**Enrichment Score: 14.813115033704484**
HNRH1_HUMAN	heterogeneous nuclear ribonucleoprotein H1 (H)(HNRNPH1)
HNRPF_HUMAN	heterogeneous nuclear ribonucleoprotein F(HNRNPF)
NUCL_HUMAN	nucleolin(NCL)
PABP1_HUMAN	poly(A) binding protein cytoplasmic 1(PABPC1)

Endosome/ER/post-translational modification

**Gene Group 6**	**Enrichment Score: 23.738299357016643**
ENOA_HUMAN	enolase 1(ENO1)
PGAM1_HUMAN	phosphoglycerate mutase 1(PGAM1)
ALDOA_HUMAN	aldolase, fructose-bisphosphate A(ALDOA)
TPIS_HUMAN	triosephosphate isomerase 1(TPI1)
PGK1_HUMAN	phosphoglycerate kinase 1(PGK1)
ENOB_HUMAN	enolase 3(ENO3)
ENOG_HUMAN	enolase 2(ENO2)
PFKAP_HUMAN	phosphofructokinase, platelet(PFKP)

**Gene Group 10**	**Enrichment Score: 20.05286787307961**
PRDX6_HUMAN	peroxiredoxin 6(PRDX6)
PRDX5_HUMAN	peroxiredoxin 5(PRDX5)
TXD17_HUMAN	thioredoxin domain containing 17(TXNDC17)
PRDX2_HUMAN	peroxiredoxin 2(PRDX2)
PRDX1_HUMAN	peroxiredoxin 1(PRDX1)

**Gene Group 11**	**Enrichment Score: 17.739344682720432**
TRXR1_HUMAN	thioredoxin reductase 1(TXNRD1)
PRDX1_HUMAN	peroxiredoxin 1(PRDX1)
TXNL1_HUMAN	thioredoxin like 1(TXNL1)
THIO_HUMAN	thioredoxin(TXN)

**Gene Group 12**	**Enrichment Score: 17.410215421211376**
PSMD2_HUMAN	proteasome 26S subunit, non-ATPase 2(PSMD2)
PSA5_HUMAN	proteasome subunit alpha 5(PSMA5)
PSMD9_HUMAN	proteasome 26S subunit, non-ATPase 9(PSMD9)
PSME1_HUMAN	proteasome activator subunit 1(PSME1)

**Gene Group 13**	**Enrichment Score: 16.230905318464163**
PDIA6_HUMAN	protein disulfide isomerase family A member 6(PDIA6)
PDIA4_HUMAN	protein disulfide isomerase family A member 4(PDIA4)
PDIA3_HUMAN	protein disulfide isomerase family A member 3(PDIA3)
PDIA1_HUMAN	prolyl 4-hydroxylase subunit beta(P4HB)
TXND5_HUMAN	thioredoxin domain containing 5(TXNDC5)

**Gene Group 17**	**Enrichment Score: 14.455028699561934**
RAB35_HUMAN	RAB35, member RAS oncogene family(RAB35)
RB11A_HUMAN	RAB11A, member RAS oncogene family(RAB11A)
RAB1A_HUMAN	RAB1A, member RAS oncogene family(RAB1A)
ARL3_HUMAN	ADP ribosylation factor like GTPase 3(ARL3)
RAB14_HUMAN	RAB14, member RAS oncogene family(RAB14)
RAB15_HUMAN	RAB15, member RAS oncogene family(RAB15)

**Gene Group 18**	**Enrichment Score: 14.389719167860317**
PP1R7_HUMAN	protein phosphatase 1 regulatory subunit 7(PPP1R7)
VASN_HUMAN	vasorin(VASN)
AN32A_HUMAN	acidic nuclear phosphoprotein 32 family member A(ANP32A)
PGS2_HUMAN	decorin(DCN)
AN32B_HUMAN	acidic nuclear phosphoprotein 32 family member B(ANP32B)
LUM_HUMAN	lumican(LUM)
RINI_HUMAN	ribonuclease/angiogenin inhibitor 1(RNH1)

**Gene Group 19**	**Enrichment Score: 12.872663885066817**
MDHC_HUMAN	malate dehydrogenase 1(MDH1)

LDHB_HUMAN	lactate dehydrogenase B(LDHB)
LDHA_HUMAN	lactate dehydrogenase A(LDHA)
MDHM_HUMAN	malate dehydrogenase 2(MDH2)

Cytoskeleton/Transport

**Gene Group 1**	**Enrichment Score: 26.06832679011804**
TAGL2_HUMAN	transgelin 2(TAGLN2)
TAGL_HUMAN	transgelin(TAGLN)
CNN3_HUMAN	calponin 3(CNN3)
CNN2_HUMAN	calponin 2(CNN2)

**Gene Group 2**	**Enrichment Score: 26.01771050622349**
FHL2_HUMAN	four and a half LIM domains 2(FHL2)
FHL1_HUMAN	four and a half LIM domains 1(FHL1)
LIMA1_HUMAN	LIM domain and actin binding 1(LIMA1)
PDLI1_HUMAN	PDZ and LIM domain 1(PDLIM1)
ZYX_HUMAN	zyxin(ZYX)
CSRP1_HUMAN	cysteine and glycine rich protein 1(CSRP1)
LASP1_HUMAN	LIM and SH3 protein 1(LASP1)

**Gene Group 3**	**Enrichment Score: 24.396715980228898**
1433Z_HUMAN	tyrosine 3-monooxygenase/tryptophan 5-monooxygenase activation protein zeta(YWHAZ)
1433F_HUMAN	tyrosine 3-monooxygenase/tryptophan 5-monooxygenase activation protein eta(YWHAH)
1433G_HUMAN	tyrosine 3-monooxygenase/tryptophan 5-monooxygenase activation protein gamma(YWHAG)
1433E_HUMAN	tyrosine 3-monooxygenase/tryptophan 5-monooxygenase activation protein epsilon(YWHAE)
1433B_HUMAN	tyrosine 3-monooxygenase/tryptophan 5-monooxygenase activation protein beta(YWHAB)
1433T_HUMAN	tyrosine 3-monooxygenase/tryptophan 5-monooxygenase activation protein theta(YWHAQ)

**Gene Group 4**	**Enrichment Score: 24.073328540106484**
TPM4_HUMAN	tropomyosin 4(TPM4)
TPM3_HUMAN	tropomyosin 3(TPM3)
TPM2_HUMAN	tropomyosin 2 (beta)(TPM2)
TPM1_HUMAN	tropomyosin 1 (alpha)(TPM1)

**Gene Group 5**	**Enrichment Score: 23.944627694232647**
ACTN1_HUMAN	actinin alpha 1(ACTN1)
PLSL_HUMAN	lymphocyte cytosolic protein 1(LCP1)
S10A6_HUMAN	S100 calcium binding protein A6(S100A6)
ACTN4_HUMAN	actinin alpha 4(ACTN4)
EFHD2_HUMAN	EF-hand domain family member D2(EFHD2)
CPNS1_HUMAN	calpain small subunit 1(CAPNS1)
PLST_HUMAN	plastin 3(PLS3)
PLSI_HUMAN	plastin 1(PLS1)

**Gene Group 7**	**Enrichment Score: 21.247460799168064**
S10A6_HUMAN	S100 calcium binding protein A6(S100A6)
CPNS1_HUMAN	calpain small subunit 1(CAPNS1)
RCN1_HUMAN	reticulocalbin 1(RCN1)
RCN3_HUMAN	reticulocalbin 3(RCN3)
EFHD2_HUMAN	EF-hand domain family member D2(EFHD2)
MYL6_HUMAN	myosin light chain 6(MYL6)
MYL1_HUMAN	myosin light chain 1(MYL1)
CALU_HUMAN	calumenin(CALU)
FKB10_HUMAN	FK506 binding protein 10(FKBP10)
MYL9_HUMAN	myosin light chain 9(MYL9)
ML12A_HUMAN	myosin light chain 12A(MYL12A)
GLU2B_HUMAN	protein kinase C substrate 80K-H(PRKCSH)

**Gene Group 8**	**Enrichment Score: 21.18293762408389**
TBB2A_HUMAN	tubulin beta 2A class IIa(TUBB2A)
TBB4B_HUMAN	tubulin beta 4B class IVb(TUBB4B)
TBA4A_HUMAN	tubulin alpha 4a(TUBA4A)
TBB3_HUMAN	tubulin beta 3 class III(TUBB3)
TBA1A_HUMAN	tubulin alpha 1a(TUBA1A)
TBA1B_HUMAN	tubulin alpha 1b(TUBA1B)
TBB5_HUMAN	tubulin beta class I(TUBB)
TBB6_HUMAN	tubulin beta 6 class V(TUBB6)
TBA4B_HUMAN	tubulin alpha 4b(TUBA4B)

**Gene Group 14**	**Enrichment Score: 15.30224348981118**
IMB1_HUMAN	karyopherin subunit beta 1(KPNB1)
XPO2_HUMAN	chromosome segregation 1 like(CSE1L)
IPO5_HUMAN	importin 5(IPO5)
TNPO1_HUMAN	transportin 1(TNPO1)
XPO1_HUMAN	exportin 1(XPO1)
IPO7_HUMAN	importin 7(IPO7)

**Gene Group 15**	**Enrichment Score: 15.06670556953282**
LMNA_HUMAN	lamin A/C(LMNA)
DESM_HUMAN	desmin(DES)
K1C10_HUMAN	keratin 10(KRT10)
K1C9_HUMAN	keratin 9(KRT9)
K2C7_HUMAN	keratin 7(KRT7)
K2C6B_HUMAN	keratin 6B(KRT6B)
K22E_HUMAN	keratin 2(KRT2)
K2C1_HUMAN	keratin 1(KRT1)
VIME_HUMAN	vimentin(VIM)

Supplementary Table 2. Result of gene functional clustering of the cytoplasmic proteome associated with PDMAEMA/PMETAC-functionalised nanoparticles. Colours indicate broad classes of functions associated with each cluster.

RNA/DNA binding / Translation

**Gene Group 2**	**Enrichment Score: 7.787899445860546**
H2A1B_HUMAN	histone cluster 1 H2A family member e(HIST1H2AE)
PA2G4_HUMAN	proliferation-associated 2G4(PA2G4)
TCP4_HUMAN	SUB1 homolog, transcriptional regulator(SUB1)
H4_HUMAN	histone cluster 4 H4(HIST4H4)
HNRPK_HUMAN	heterogeneous nuclear ribonucleoprotein K(HNRNPK)
NC2B_HUMAN	down-regulator of transcription 1(DR1)
HDGF_HUMAN	hepatoma-derived growth factor(HDGF)
HGB1A_HUMAN	high mobility group box 1 pseudogene 1(HMGB1P1)
SARNP_HUMAN	SAP domain containing ribonucleoprotein(SARNP)
H2B1B_HUMAN	histone cluster 1 H2B family member b(HIST1H2BB)
YBOX1_HUMAN	Y-box binding protein 1(YBX1)
H2B1J_HUMAN	histone cluster 1 H2B family member j(HIST1H2BJ)

Endosome/ER/post-translational modification

**Gene Group 1**	**Enrichment Score: 16.609619652141046**
HEBP2_HUMAN	heme binding protein 2(HEBP2)
PP1RB_HUMAN	protein phosphatase 1 regulatory inhibitor subunit 11(PPP1R11)
PPR18_HUMAN	protein phosphatase 1 regulatory subunit 18(PPP1R18)

**Gene Group 6**	**Enrichment Score: 3.338523045428616**
CALU_HUMAN	calumenin(CALU)
NUCB1_HUMAN	nucleobindin 1(NUCB1)
MYL6_HUMAN	myosin light chain 6(MYL6)
RCN1_HUMAN	reticulocalbin 1(RCN1)
ML12A_HUMAN	myosin light chain 12A(MYL12A)

Cytoskeleton/Transport

**Gene Group 3**	**Enrichment Score: 7.693520578901487**
LIMA1_HUMAN	LIM domain and actin binding 1(LIMA1)
PDLI1_HUMAN	PDZ and LIM domain 1(PDLIM1)
ZYX_HUMAN	zyxin(ZYX)
LASP1_HUMAN	LIM and SH3 protein 1(LASP1)

**Gene Group 4**	**Enrichment Score: 5.719346211530507**
MAP4_HUMAN	microtubule associated protein 4(MAP4)
MAP1A_HUMAN	microtubule associated protein 1A(MAP1A)
NDRG1_HUMAN	N-myc downstream regulated 1(NDRG1)

**Gene Group 5**	**Enrichment Score: 4.726289691527206**
K22E_HUMAN	keratin 2(KRT2)
K2C1_HUMAN	keratin 1(KRT1)
LMNA_HUMAN	lamin A/C(LMNA)
VIME_HUMAN	vimentin(VIM)

Supplementary Table 3. Result of gene functional clustering of the cell lysate proteome associated with PDMAEMA-functionalised nanoparticles. Colours indicate broad classes of functions associated with each cluster.

RNA/DNA binding / Transcription

**Gene Group 1**	**Enrichment Score: 30.294652091307988**
EIF3A_HUMAN	eukaryotic translation initiation factor 3 subunit A(EIF3A)
EIF3B_HUMAN	eukaryotic translation initiation factor 3 subunit B(EIF3B)
EIF3C_HUMAN	eukaryotic translation initiation factor 3 subunit C(EIF3C)
EIF3J_HUMAN	eukaryotic translation initiation factor 3 subunit J(EIF3J)
EIF3L_HUMAN	eukaryotic translation initiation factor 3 subunit L(EIF3L)
IF2P_HUMAN	eukaryotic translation initiation factor 5B(EIF5B)

**Gene Group 2**	**Enrichment Score: 29.131006738267395**
ACINU_HUMAN	apoptotic chromatin condensation inducer 1(ACIN1)
BCLF1_HUMAN	BCL2 associated transcription factor 1(BCLAF1)
CCAR1_HUMAN	cell division cycle and apoptosis regulator 1(CCAR1)
CCAR2_HUMAN	cell cycle and apoptosis regulator 2(CCAR2)
LSM2_HUMAN	LSM2 homolog, U6 small nuclear RNA and mRNA degradation associated(LSM2)
PININ_HUMAN	pinin, desmosome associated protein(PNN)
PP4R2_HUMAN	protein phosphatase 4 regulatory subunit 2(PPP4R2)
PR38A_HUMAN	pre-mRNA processing factor 38A(PRPF38A)
PRP31_HUMAN	pre-mRNA processing factor 31(PRPF31)
RBM25_HUMAN	RNA binding motif protein 25(RBM25)
RBM8A_HUMAN	RNA binding motif protein 8A(RBM8A)
RUXF_HUMAN	small nuclear ribonucleoprotein polypeptide F(SNRPF)
SCAF8_HUMAN	SR-related CTD associated factor 8(SCAF8)
SCAFB_HUMAN	SR-related CTD associated factor 11(SCAF11)
SF3A3_HUMAN	splicing factor 3a subunit 3(SF3A3)
SF3B1_HUMAN	splicing factor 3b subunit 1(SF3B1)
SF3B2_HUMAN	splicing factor 3b subunit 2(SF3B2)
SF3B3_HUMAN	splicing factor 3b subunit 3(SF3B3)
SMD1_HUMAN	small nuclear ribonucleoprotein D1 polypeptide(SNRPD1)
SRRM1_HUMAN	serine and arginine repetitive matrix 1(SRRM1)
SRRM2_HUMAN	serine/arginine repetitive matrix 2(SRRM2)
U2AF2_HUMAN	U2 small nuclear RNA auxiliary factor 2(U2AF2)

**Gene Group 6**	**Enrichment Score: 27.655756536070797**
SYCC_HUMAN	cysteinyl-tRNA synthetase(CARS)
SYDC_HUMAN	aspartyl-tRNA synthetase(DARS)
SYEP_HUMAN	glutamyl-prolyl-tRNA synthetase(EPRS)
SYIM_HUMAN	isoleucyl-tRNA synthetase 2, mitochondrial(IARS2)
SYK_HUMAN	lysyl-tRNA synthetase(KARS)
SYNM_HUMAN	asparaginyl-tRNA synthetase 2, mitochondrial (putative)(NARS2)
SYQ_HUMAN	glutaminyl-tRNA synthetase(QARS)
SYVC_HUMAN	valyl-tRNA synthetase(VARS)
SYYC_HUMAN	tyrosyl-tRNA synthetase(YARS)

**Gene Group 8**	**Enrichment Score: 26.471663060087685**
ACINU_HUMAN	apoptotic chromatin condensation inducer 1(ACIN1)
CPSF7_HUMAN	cleavage and polyadenylation specific factor 7(CPSF7)
DX39B_HUMAN	DExD-box helicase 39B(DDX39B)
G3BP1_HUMAN	G3BP stress granule assembly factor 1(G3BP1)
HNRLL_HUMAN	heterogeneous nuclear ribonucleoprotein L like(HNRNPLL)
HNRPR_HUMAN	heterogeneous nuclear ribonucleoprotein R(HNRNPR)
LARP7_HUMAN	La ribonucleoprotein domain family member 7(LARP7)
NXF1_HUMAN	nuclear RNA export factor 1(NXF1)
PABP2_HUMAN	poly(A) binding protein nuclear 1(PABPN1)
PPIL4_HUMAN	peptidylprolyl isomerase like 4(PPIL4)
PUF60_HUMAN	poly(U) binding splicing factor 60(PUF60)
RBM25_HUMAN	RNA binding motif protein 25(RBM25)
RBM39_HUMAN	RNA binding motif protein 39(RBM39)
RBM8A_HUMAN	RNA binding motif protein 8A(RBM8A)
SAFB2_HUMAN	scaffold attachment factor B2(SAFB2)
SART3_HUMAN	squamous cell carcinoma antigen recognized by T-cells 3(SART3)
SCAF8_HUMAN	SR-related CTD associated factor 8(SCAF8)
SF3B2_HUMAN	splicing factor 3b subunit 2(SF3B2)
SR140_HUMAN	U2 snRNP associated SURP domain containing(U2SURP)
SRRM1_HUMAN	serine and arginine repetitive matrix 1(SRRM1)
SRSF4_HUMAN	serine and arginine rich splicing factor 4(SRSF4)
SRSF6_HUMAN	serine and arginine rich splicing factor 6(SRSF6)
THOC1_HUMAN	THO complex 1(THOC1)
THOC2_HUMAN	THO complex 2(THOC2)
U2AF2_HUMAN	U2 small nuclear RNA auxiliary factor 2(U2AF2)
YTDC1_HUMAN	YTH domain containing 1(YTHDC1)
ZN638_HUMAN	zinc finger protein 638(ZNF638)

**Gene Group 9**	**Enrichment Score: 26.16811080411271**
BCLF1_HUMAN	BCL2 associated transcription factor 1(BCLAF1)
CCAR1_HUMAN	cell division cycle and apoptosis regulator 1(CCAR1)
LRRF1_HUMAN	LRR binding FLII interacting protein 1(LRRFIP1)
MBB1A_HUMAN	MYB binding protein 1a(MYBBP1A)
PA2G4_HUMAN	proliferation-associated 2G4(PA2G4)
PURA_HUMAN	purine rich element binding protein A(PURA)
PURB_HUMAN	purine rich element binding protein B(PURB)
SAFB2_HUMAN	scaffold attachment factor B2(SAFB2)
SRRT_HUMAN	serrate, RNA effector molecule(SRRT)
YBOX1_HUMAN	Y-box binding protein 1(YBX1)

**Gene Group 11**	**Enrichment Score: 24.938757027378568**
ASCC3_HUMAN	activating signal cointegrator 1 complex subunit 3(ASCC3)
ATRX_HUMAN	ATRX, chromatin remodeler(ATRX)
CHD4_HUMAN	chromodomain helicase DNA binding protein 4(CHD4)
DDX1_HUMAN	DEAD-box helicase 1(DDX1)
DDX18_HUMAN	DEAD-box helicase 18(DDX18)
DDX23_HUMAN	DEAD-box helicase 23(DDX23)
DDX24_HUMAN	DEAD-box helicase 24(DDX24)
DDX3X_HUMAN	DEAD-box helicase 3, X-linked(DDX3X)
DDX46_HUMAN	DEAD-box helicase 46(DDX46)
DDX54_HUMAN	DEAD-box helicase 54(DDX54)

DDX6_HUMAN DEAD-box helicase 6(DDX6)

DDX60_HUMAN DExD/H-box helicase 60(DDX60)

DHX16_HUMAN DEAH-box helicase 16(DHX16)

DHX29_HUMAN DExH-box helicase 29(DHX29)

DHX30_HUMAN DExH-box helicase 30(DHX30)

DHX36_HUMAN DEAH-box helicase 36(DHX36)

DHX8_HUMAN DEAH-box helicase 8(DHX8)

DX39B_HUMAN DExD-box helicase 39B(DDX39B)

IF4A3_HUMAN eukaryotic translation initiation factor 4A3(EIF4A3)

IFIH1_HUMAN interferon induced with helicase C domain 1(IFIH1)

RECQ1_HUMAN RecQ like helicase(RECQL)

SMCA1_HUMAN SWI/SNF related, matrix associated, actin dependent regulator of chromatin, subfamily a, member 1(SMARCA1)

SMCA2_HUMAN SWI/SNF related, matrix associated, actin dependent regulator of chromatin, subfamily a, member 2(SMARCA2)

SMCA4_HUMAN SWI/SNF related, matrix associated, actin dependent regulator of chromatin, subfamily a, member 4(SMARCA4)

SMCA5_HUMAN SWI/SNF related, matrix associated, actin dependent regulator of chromatin, subfamily a, member 5(SMARCA5)

SMRC2_HUMAN SWI/SNF related, matrix associated, actin dependent regulator of chromatin subfamily c member 2(SMARCC2)

U520_HUMAN small nuclear ribonucleoprotein U5 subunit 200(SNRNP200)

Gene Group 12 Enrichment Score: 24.325512600165624

IMA1_HUMAN karyopherin subunit alpha 2(KPNA2)

IMA4_HUMAN karyopherin subunit alpha 3(KPNA3)

IPO5_HUMAN importin 5(IPO5)

IPO7_HUMAN importin 7(IPO7)

IPO9_HUMAN importin 9(IPO9)

TNPO1_HUMAN transportin 1(TNPO1)

XPO1_HUMAN exportin 1(XPO1)

Gene Group 17 Enrichment Score: 22.773899013009572

CND1_HUMAN non-SMC condensin I complex subunit D2(NCAPD2)

PDS5A_HUMAN PDS5 cohesin associated factor A(PDS5A)

PDS5B_HUMAN PDS5 cohesin associated factor B(PDS5B)

RAD21_HUMAN RAD21 cohesin complex component(RAD21)

SMC1A_HUMAN structural maintenance of chromosomes 1A(SMC1A)

SMC3_HUMAN structural maintenance of chromosomes 3(SMC3)

SMC4_HUMAN structural maintenance of chromosomes 4(SMC4)

STAG1_HUMAN stromal antigen 1(STAG1)

STAG2_HUMAN stromal antigen 2(STAG2)

Gene Group 19 Enrichment Score: 22.24640367816701

CAND1_HUMAN cullin associated and neddylation dissociated 1(CAND1)

DAAF5_HUMAN dynein axonemal assembly factor 5(DNAAF5)

ECM29_HUMAN KIAA0368(KIAA0368)

PP4R1_HUMAN protein phosphatase 4 regulatory subunit 1(PPP4R1)

SF3B1_HUMAN splicing factor 3b subunit 1(SF3B1)

Gene Group 23 Enrichment Score: 19.4776871730492

NU107_HUMAN nucleoporin 107(NUP107)

NU133_HUMAN nucleoporin 133(NUP133)

NU155_HUMAN nucleoporin 155(NUP155)

NU160_HUMAN nucleoporin 160(NUP160)

NUP93_HUMAN nucleoporin 93(NUP93)

NUP98_HUMAN	nucleoporin 98(NUP98)
P121C_HUMAN	POM121 transmembrane nucleoporin C(POM121C)
RBP2_HUMAN	RAN binding protein 2(RANBP2)
TPR_HUMAN	translocated promoter region, nuclear basket protein(TPR)

**Gene Group 24**	**Enrichment Score: 18.888810510573265**
RL17_HUMAN	ribosomal protein L17(RPL17)
RL35A_HUMAN	ribosomal protein L35a(RPL35A)
RLA0_HUMAN	ribosomal protein lateral stalk subunit P0(RPLP0)
RS10_HUMAN	ribosomal protein S10(RPS10)
RS17_HUMAN	ribosomal protein S17(RPS17)
RS26_HUMAN	40S ribosomal protein S26(LOC101929876)
RS27_HUMAN	ribosomal protein S27(RPS27)
RS27L_HUMAN	ribosomal protein S27 like(RPS27L)

**Gene Group 25**	**Enrichment Score: 18.540431858160034**
MPP10_HUMAN	M-phase phosphoprotein 10(MPHOSPH10)
NOP14_HUMAN	NOP14 nucleolar protein(NOP14)
NOP56_HUMAN	NOP56 ribonucleoprotein(NOP56)
NOP58_HUMAN	NOP58 ribonucleoprotein(NOP58)

**Gene Group 30**	**Enrichment Score: 16.315592030058088**
BOP1_HUMAN	block of proliferation 1(BOP1)
EDC4_HUMAN	enhancer of mRNA decapping 4(EDC4)
PRP19_HUMAN	pre-mRNA processing factor 19(PRPF19)
PWP1_HUMAN	PWP1 homolog, endonuclein(PWP1)
PWP2_HUMAN	periodic tryptophan protein 2 homolog(LOC102724159)
RBBP4_HUMAN	RB binding protein 4, chromatin remodeling factor(RBBP4)
RBBP7_HUMAN	RB binding protein 7, chromatin remodeling factor(RBBP7)
STRAP_HUMAN	serine/threonine kinase receptor associated protein(STRAP)
STRN_HUMAN	striatin(STRN)
STRN3_HUMAN	striatin 3(STRN3)
WDR11_HUMAN	WD repeat domain 11(WDR11)
WDR43_HUMAN	WD repeat domain 43(WDR43)
WDR44_HUMAN	WD repeat domain 44(WDR44)

**Gene Group 31**	**Enrichment Score: 16.13094728827634**
ASCC3_HUMAN	activating signal cointegrator 1 complex subunit 3(ASCC3)
MCM2_HUMAN	minichromosome maintenance complex component 2(MCM2)
MCM3_HUMAN	minichromosome maintenance complex component 3(MCM3)
MCM6_HUMAN	minichromosome maintenance complex component 6(MCM6)
MCM7_HUMAN	minichromosome maintenance complex component 7(MCM7)
RECQ1_HUMAN	RecQ like helicase(RECQL)

**Gene Group 33**	**Enrichment Score: 14.495528770522379**
EHD1_HUMAN	EH domain containing 1(EHD1)
EHD3_HUMAN	EH domain containing 3(EHD3)
EHD4_HUMAN	EH domain containing 4(EHD4)
EP15R_HUMAN	epidermal growth factor receptor pathway substrate 15 like 1(EPS15L1)

**Gene Group 37**	**Enrichment Score: 10.481218958784016**
ARF1_HUMAN	ADP ribosylation factor 1(ARF1)

ARF5_HUMAN	ADP ribosylation factor 5(ARF5)
ARF6_HUMAN	ADP ribosylation factor 6(ARF6)
ARL1_HUMAN	ADP ribosylation factor like GTPase 1(ARL1)
ARL8A_HUMAN	ADP ribosylation factor like GTPase 8A(ARL8A)
ARL8B_HUMAN	ADP ribosylation factor like GTPase 8B(ARL8B)
GBP2_HUMAN	guanylate binding protein 2(GBP2)
RAB10_HUMAN	RAB10, member RAS oncogene family(RAB10)
RAB18_HUMAN	RAB18, member RAS oncogene family(RAB18)
RAB1A_HUMAN	RAB1A, member RAS oncogene family(RAB1A)
RAB21_HUMAN	RAB21, member RAS oncogene family(RAB21)
RAB8A_HUMAN	RAB8A, member RAS oncogene family(RAB8A)
RB11B_HUMAN	RAB11B, member RAS oncogene family(RAB11B)
RHOG_HUMAN	ras homolog family member G(RHOG)
RRAS_HUMAN	related RAS viral (r-ras) oncogene homolog(RRAS)
RRAS2_HUMAN	related RAS viral (r-ras) oncogene homolog 2(RRAS2)
SAR1A_HUMAN	secretion associated Ras related GTPase 1A(SAR1A)
SEP10_HUMAN	septin 10(SEPT10)

**Gene Group 5**	**Enrichment Score: 28.495045479716975**
COG5_HUMAN	component of oligomeric golgi complex 5(COG5)
COPD_HUMAN	archain 1(ARCN1)
COPE_HUMAN	coatomer protein complex subunit epsilon(COPE)
COPZ1_HUMAN	coatomer protein complex subunit zeta 1(COPZ1)
COPZ2_HUMAN	coatomer protein complex subunit zeta 2(COPZ2)
GOGA3_HUMAN	golgin A3(GOLGA3)
OSBP1_HUMAN	oxysterol binding protein(OSBP)
SC24A_HUMAN	SEC24 homolog A, COPII coat complex component(SEC24A)
SCFD1_HUMAN	sec1 family domain containing 1(SCFD1)
YKT6_HUMAN	YKT6 v-SNARE homolog (S. cerevisiae)(YKT6)

**Gene Group 21**	**Enrichment Score: 21.056209137761673**
ABCE1_HUMAN	ATP binding cassette subfamily E member 1(ABCE1)
ABCF1_HUMAN	ATP binding cassette subfamily F member 1(ABCF1)
ABCF2_HUMAN	ATP binding cassette subfamily F member 2(ABCF2)
MRP4_HUMAN	ATP binding cassette subfamily C member 4(ABCC4)

Endosome / Proteasome / Exocytosis

**Gene Group 7**	**Enrichment Score: 27.452478515212295**
KANK2_HUMAN	KN motif and ankyrin repeat domains 2(KANK2)
OSTF1_HUMAN	osteoclast stimulating factor 1(OSTF1)
POTEE_HUMAN	POTE ankyrin domain family member E(POTEE)
RAI14_HUMAN	retinoic acid induced 14(RAI14)

**Gene Group 3**	**Enrichment Score: 28.83726348296347**
EXOC1_HUMAN	exocyst complex component 1(EXOC1)
EXOC4_HUMAN	exocyst complex component 4(EXOC4)
EXOC5_HUMAN	exocyst complex component 5(EXOC5)
EXOC7_HUMAN	exocyst complex component 7(EXOC7)
EXOC8_HUMAN	exocyst complex component 8(EXOC8)

**Gene Group 13**	**Enrichment Score: 24.092753945374465**

AMPB_HUMAN arginyl aminopeptidase(RNPEP)
AMPL_HUMAN leucine aminopeptidase 3(LAP3)
CNDP2_HUMAN CNDP dipeptidase 2 (metallopeptidase M20 family)(CNDP2)
DNPEP_HUMAN aspartyl aminopeptidase(DNPEP)
DPP3_HUMAN dipeptidyl peptidase 3(DPP3)
LKHA4_HUMAN leukotriene A4 hydrolase(LTA4H)
XPP1_HUMAN X-prolyl aminopeptidase 1(XPNPEP1)

Gene Group 14 Enrichment Score: 23.991355271399687
ANO10_HUMAN anoctamin 10(ANO10)
ARMX2_HUMAN armadillo repeat containing, X-linked 2(ARMCX2)
AT11C_HUMAN ATPase phospholipid transporting 11C(ATP11C)
ATLA3_HUMAN atlastin GTPase 3(ATL3)
CCD51_HUMAN coiled-coil domain containing 51(CCDC51)
CLPT1_HUMAN CLPTM1, transmembrane protein(CLPTM1)
EMC3_HUMAN ER membrane protein complex subunit 3(EMC3)
F162A_HUMAN family with sequence similarity 162 member A(FAM162A)
GGT7_HUMAN gamma-glutamyltransferase 7(GGT7)
GLPC_HUMAN glycophorin C (Gerbich blood group)(GYPC)
GOGB1_HUMAN golgin B1(GOLGB1)
GOLI4_HUMAN golgi integral membrane protein 4(GOLIM4)
HM13_HUMAN histocompatibility minor 13(HM13)
KIRR1_HUMAN kin of IRRE like (Drosophila)(KIRREL)
LMAN1_HUMAN lectin, mannose binding 1(LMAN1)
LMAN2_HUMAN lectin, mannose binding 2(LMAN2)
LMBD2_HUMAN LMBR1 domain containing 2(LMBRD2)
MLEC_HUMAN malectin(MLEC)
MOGS_HUMAN mannosyl-oligosaccharide glucosidase(MOGS)
MOT1_HUMAN solute carrier family 16 member 1(SLC16A1)
MOT4_HUMAN solute carrier family 16 member 3(SLC16A3)
MTCH2_HUMAN mitochondrial carrier 2(MTCH2)
MTX1_HUMAN metaxin 1(MTX1)
NOMO2_HUMAN NODAL modulator 2(NOMO2)
PBIP1_HUMAN PBX homeobox interacting protein 1(PBXIP1)
PIGS_HUMAN phosphatidylinositol glycan anchor biosynthesis class S(PIGS)
PLPL6_HUMAN patatin like phospholipase domain containing 6(PNPLA6)
PODXL_HUMAN podocalyxin like(PODXL)
PRAF3_HUMAN ADP ribosylation factor like GTPase 6 interacting protein 5(ARL6IP5)
REEP3_HUMAN receptor accessory protein 3(REEP3)
RMD3_HUMAN regulator of microtubule dynamics 3(RMDN3)
S12A9_HUMAN solute carrier family 12 member 9(SLC12A9)
S38AA_HUMAN solute carrier family 38 member 10(SLC38A10)
SAM50_HUMAN SAMM50 sorting and assembly machinery component(SAMM50)
SCAM1_HUMAN secretory carrier membrane protein 1(SCAMP1)
SCAM3_HUMAN secretory carrier membrane protein 3(SCAMP3)
SE1L1_HUMAN SEL1L ERAD E3 ligase adaptor subunit(SEL1L)
SSRD_HUMAN signal sequence receptor subunit 4(SSR4)
STBD1_HUMAN starch binding domain 1(STBD1)
STT3B_HUMAN STT3B, catalytic subunit of the oligosaccharyltransferase complex(STT3B)
SURF4_HUMAN surfeit 4(SURF4)
T184C_HUMAN transmembrane protein 184C(TMEM184C)
TECR_HUMAN trans-2,3-enoyl-CoA reductase(TECR)

TGON2_HUMAN trans-golgi network protein 2(TGOLN2)
TIDC1_HUMAN translocase of inner mitochondrial membrane domain containing 1(TIMMDC1)

TM119_HUMAN transmembrane protein 119(TMEM119)

TM87A_HUMAN transmembrane protein 87A(TMEM87A)

TMM51_HUMAN transmembrane protein 51(TMEM51)

TMUB1_HUMAN transmembrane and ubiquitin like domain containing 1(TMUB1)

TMX1_HUMAN thioredoxin related transmembrane protein 1(TMX1)

TMX2_HUMAN thioredoxin related transmembrane protein 2(TMX2)

TOIP1_HUMAN torsin 1A interacting protein 1(TOR1AIP1)

TOIP2_HUMAN torsin 1A interacting protein 2(TOR1AIP2)

VMP1_HUMAN vacuole membrane protein 1(VMP1)

YIPF6_HUMAN Yip1 domain family member 6(YIPF6)

ZDHC5_HUMAN zinc finger DHHC-type containing 5(ZDHHC5)

Gene Group 15 Enrichment Score: 23.560138697961047

EMC2_HUMAN ER membrane protein complex subunit 2(EMC2)

IFIT1_HUMAN interferon induced protein with tetratricopeptide repeats 1(IFIT1)

IFIT2_HUMAN interferon induced protein with tetratricopeptide repeats 2(IFIT2)

NAA15_HUMAN N(alpha)-acetyltransferase 15, NatA auxiliary subunit(NAA15)

SRP72_HUMAN signal recognition particle 72(SRP72)

TOM70_HUMAN translocase of outer mitochondrial membrane 70(TOMM70)

TTC37_HUMAN tetratricopeptide repeat domain 37(TTC37)

Gene Group 16 Enrichment Score: 23.32386090669039

ADDG_HUMAN adducin 3(ADD3)

MPRIP_HUMAN myosin phosphatase Rho interacting protein(MPRIP)

PPR18_HUMAN protein phosphatase 1 regulatory subunit 18(PPP1R18)

SNTB1_HUMAN syntrophin beta 1(SNTB1)

Gene Group 18 Enrichment Score: 22.47396005842723

DOC10_HUMAN dedicator of cytokinesis 10(DOCK10)

DOCK5_HUMAN dedicator of cytokinesis 5(DOCK5)

DOCK7_HUMAN dedicator of cytokinesis 7(DOCK7)

DOCK8_HUMAN dedicator of cytokinesis 8(DOCK8)

Gene Group 26 Enrichment Score: 17.97185046387675

SCAM1_HUMAN secretory carrier membrane protein 1(SCAMP1)

SNP23_HUMAN synaptosome associated protein 23(SNAP23)

STX12_HUMAN syntaxin 12(STX12)

STX2_HUMAN syntaxin 2(STX2)

STX3_HUMAN syntaxin 3(STX3)

STX6_HUMAN syntaxin 6(STX6)

STX7_HUMAN syntaxin 7(STX7)

VTI1A_HUMAN vesicle transport through interaction with t-SNAREs 1A(VTI1A)

VTI1B_HUMAN vesicle transport through interaction with t-SNAREs 1B(VTI1B)

Gene Group 27 Enrichment Score: 17.83828446499592

BMP2K_HUMAN BMP2 inducible kinase(BMP2K)

KS6A3_HUMAN ribosomal protein S6 kinase A3(RPS6KA3)

M3K7_HUMAN mitogen-activated protein kinase kinase kinase 7(MAP3K7)

M4K4_HUMAN mitogen-activated protein kinase kinase kinase kinase 4(MAP4K4)

MP2K3_HUMAN mitogen-activated protein kinase kinase 3(MAP2K3)
MRCKA_HUMAN CDC42 binding protein kinase alpha(CDC42BPA)
MRCKB_HUMAN CDC42 binding protein kinase beta(CDC42BPB)
NEK9_HUMAN NIMA related kinase 9(NEK9)
NRBP2_HUMAN nuclear receptor binding protein 2(NRBP2)
PAK2_HUMAN p21 (RAC1) activated kinase 2(PAK2)
PI3R4_HUMAN phosphoinositide-3-kinase regulatory subunit 4(PIK3R4)
PKN2_HUMAN protein kinase N2(PKN2)
ROCK1_HUMAN Rho associated coiled-coil containing protein kinase 1(ROCK1)
ROCK2_HUMAN Rho associated coiled-coil containing protein kinase 2(ROCK2)
SLK_HUMAN STE20 like kinase(SLK)
SRPK2_HUMAN SRSF protein kinase 2(SRPK2)
STK10_HUMAN serine/threonine kinase 10(STK10)
STK3_HUMAN serine/threonine kinase 3(STK3)
STK39_HUMAN serine/threonine kinase 39(STK39)
STK4_HUMAN serine/threonine kinase 4(STK4)
TNIK_HUMAN TRAF2 and NCK interacting kinase(TNIK)

Gene Group 28 Enrichment Score: 17.581467282337076
PRS10_HUMAN proteasome 26S subunit, ATPase 6(PSMC6)
PRS6B_HUMAN proteasome 26S subunit, ATPase 4(PSMC4)
PSD11_HUMAN proteasome 26S subunit, non-ATPase 11(PSMD11)
PSD12_HUMAN proteasome 26S subunit, non-ATPase 12(PSMD12)
PSD13_HUMAN proteasome 26S subunit, non-ATPase 13(PSMD13)
PSMD1_HUMAN proteasome 26S subunit, non-ATPase 1(PSMD1)
PSMD5_HUMAN proteasome 26S subunit, non-ATPase 5(PSMD5)
PSME1_HUMAN proteasome activator subunit 1(PSME1)
PSME2_HUMAN proteasome activator subunit 2(PSME2)

Gene Group 29 Enrichment Score: 16.765672413056556
RBGP1_HUMAN RAB GTPase activating protein 1(RABGAP1)
TB10B_HUMAN TBC1 domain family member 10B(TBC1D10B)
TBC17_HUMAN TBC1 domain family member 17(TBC1D17)
TBCD5_HUMAN TBC1 domain family member 5(TBC1D5)

Gene Group 38 Enrichment Score: 10.29828847476302
AP1B1_HUMAN adaptor related protein complex 1 beta 1 subunit(AP1B1)
AP1G1_HUMAN adaptor related protein complex 1 gamma 1 subunit(AP1G1)
AP2A1_HUMAN adaptor related protein complex 2 alpha 1 subunit(AP2A1)
AP2A2_HUMAN adaptor related protein complex 2 alpha 2 subunit(AP2A2)
AP3D1_HUMAN adaptor related protein complex 3 delta 1 subunit(AP3D1)

Gene Group 39 Enrichment Score: 10.275879061786087
VATC1_HUMAN ATPase H+ transporting V1 subunit C1(ATP6V1C1)
VATD_HUMAN ATPase H+ transporting V1 subunit D(ATP6V1D)
VATE1_HUMAN ATPase H+ transporting V1 subunit E1(ATP6V1E1)
VATH_HUMAN ATPase H+ transporting V1 subunit H(ATP6V1H)
VPP1_HUMAN ATPase H+ transporting V0 subunit a1(ATP6V0A1)
VPP3_HUMAN T-cell immune regulator 1, ATPase H+ transporting V0 subunit a3(TCIRG1)

Gene Group 40 Enrichment Score: 8.853055859693885
BASI_HUMAN basigin (Ok blood group)(BSG)

CD166_HUMAN activated leukocyte cell adhesion molecule(ALCAM)
CD276_HUMAN CD276 molecule(CD276)
KIRR1_HUMAN kin of IRRE like (Drosophila)(KIRREL)
LRC4B_HUMAN leucine rich repeat containing 4B(LRRC4B)
NOMO2_HUMAN NODAL modulator 2(NOMO2)
NPTN_HUMAN neuroplastin(NPTN)

Gene Group 41 Enrichment Score: 5.995642667401202
VP33A_HUMAN VPS33A, CORVET/HOPS core subunit(VPS33A)
VPS11_HUMAN VPS11, CORVET/HOPS core subunit(VPS11)
VPS16_HUMAN VPS16, CORVET/HOPS core subunit(VPS16)
VPS39_HUMAN VPS39, HOPS complex subunit(VPS39)

Gene Group 35 Enrichment Score: 12.37677056455638
CETN2_HUMAN centrin 2(CETN2)
CETN3_HUMAN centrin 3(CETN3)
FKB10_HUMAN FK506 binding protein 10(FKBP10)
HPCL1_HUMAN hippocalcin like 1(HPCAL1)
MYL9_HUMAN myosin light chain 9(MYL9)
RCN2_HUMAN reticulocalbin 2(RCN2)

Gene Group 20 Enrichment Score: 21.54982274441014
GDIB_HUMAN GDP dissociation inhibitor 2(GDI2)
RHG01_HUMAN Rho GTPase activating protein 1(ARHGAP1)
RHG12_HUMAN Rho GTPase activating protein 12(ARHGAP12)
RHG17_HUMAN Rho GTPase activating protein 17(ARHGAP17)
RHG18_HUMAN Rho GTPase activating protein 18(ARHGAP18)

Gene Group 10 Enrichment Score: 25.451963586842773
ATD3A_HUMAN ATPase family, AAA domain containing 3A(ATAD3A)
ATP5I_HUMAN ATP synthase, H+ transporting, mitochondrial Fo complex subunit E(ATP5I)
CCD51_HUMAN coiled-coil domain containing 51(CCDC51)
CDS2_HUMAN CDP-diacylglycerol synthase 2(CDS2)
LETM1_HUMAN leucine zipper and EF-hand containing transmembrane protein 1(LETM1)
MCU_HUMAN mitochondrial calcium uniporter(MCU)
MTCH2_HUMAN mitochondrial carrier 2(MTCH2)
NDUA9_HUMAN NADH:ubiquinone oxidoreductase subunit A9(NDUFA9)
NDUB4_HUMAN NADH:ubiquinone oxidoreductase subunit B4(NDUFB4)
NDUB6_HUMAN NADH:ubiquinone oxidoreductase subunit B6(NDUFB6)
NDUBA_HUMAN NADH:ubiquinone oxidoreductase subunit B10(NDUFB10)
OXA1L_HUMAN OXA1L, mitochondrial inner membrane protein(OXA1L)
TIDC1_HUMAN translocase of inner mitochondrial membrane domain containing 1(TIMMDC1)

Gene Group 36 Enrichment Score: 11.019993500411852
ADH1B_HUMAN alcohol dehydrogenase 1B (class I), beta polypeptide(ADH1B)
ADH1G_HUMAN alcohol dehydrogenase 1C (class I), gamma polypeptide(ADH1C)
MECR_HUMAN mitochondrial trans-2-enoyl-CoA reductase(MECR)
VAT1L_HUMAN vesicle amine transport 1 like(VAT1L)

Mitochondria

**Gene Group 32**	**Enrichment Score: 14.567371531323086**
RM19_HUMAN	mitochondrial ribosomal protein L19(MRPL19)
RM39_HUMAN	mitochondrial ribosomal protein L39(MRPL39)
RM46_HUMAN	mitochondrial ribosomal protein L46(MRPL46)
RT05_HUMAN	mitochondrial ribosomal protein S5(MRPS5)
RT22_HUMAN	mitochondrial ribosomal protein S22(MRPS22)
RT34_HUMAN	mitochondrial ribosomal protein S34(MRPS34)

Cytoskeleton

**Gene Group 34**	**Enrichment Score: 13.476680470308908**
TBA1A_HUMAN	tubulin alpha 1a(TUBA1A)
TBA1C_HUMAN	tubulin alpha 1c(TUBA1C)
TBB3_HUMAN	tubulin beta 3 class III(TUBB3)
TBB4A_HUMAN	tubulin beta 4A class IVa(TUBB4A)
TBB6_HUMAN	tubulin beta 6 class V(TUBB6)

**Gene Group 4**	**Enrichment Score: 28.561044957353644**
DREB_HUMAN	drebrin 1(DBN1)
LIMA1_HUMAN	LIM domain and actin binding 1(LIMA1)
MPRIP_HUMAN	myosin phosphatase Rho interacting protein(MPRIP)
TWF1_HUMAN	twinfilin actin binding protein 1(TWF1)

**Gene Group 22**	**Enrichment Score: 19.52496786756166**
MYO1B_HUMAN	myosin IB(MYO1B)
MYO1C_HUMAN	myosin IC(MYO1C)
MYO1D_HUMAN	myosin ID(MYO1D)
MYO1E_HUMAN	myosin IE(MYO1E)
MYO5A_HUMAN	myosin VA(MYO5A)
MYO6_HUMAN	myosin VI(MYO6)
MYO9B_HUMAN	myosin IXB(MYO9B)

ECM

**Gene Group 42**	**Enrichment Score: 3.504215914966366**
ITA2_HUMAN	integrin subunit alpha 2(ITGA2)
ITA3_HUMAN	integrin subunit alpha 3(ITGA3)
ITAV_HUMAN	integrin subunit alpha V(ITGAV)
ITB5_HUMAN	integrin subunit beta 5(ITGB5)

**Gene Group 43**	**Enrichment Score: 2.8867936435715107**
CO5A1_HUMAN	collagen type V alpha 1 chain(COL5A1)
CO6A2_HUMAN	collagen type VI alpha 2 chain(COL6A2)
COIA1_HUMAN	collagen type XVIII alpha 1 chain(COL18A1)
EMIL1_HUMAN	elastin microfibril interfacer 1(EMILIN1)
LAMA4_HUMAN	laminin subunit alpha 4(LAMA4)

Supplementary Table 4. Bromine, carbon, iodine, nitrogen, oxygen content (atom percentage) measured by XPS for PDMAEMA, PMETAC and CS-PMETAC brushes.

	Survey to atom%					High resolution				
						C*1s* %			N*1s* %	
	Br	C	I	N	O	O-C=O	C=OH, C-O-C	C-C	C-N+	C-N
PDMAEMA	-	73.7	-	8.0	18.3	8.1	15.4	45.2	0.9	6.7
PMETAC	-	71.0	7.1	6.2	15.7	5.8	37.3	25.8	6.4	0.5
CS-PMETAC	6.3	69.3	-	7.1	17.3	8.1	32.3	28.3	6.5	0.8

Supplementary Table 5. Summary of statistical analysis for HaCaT cell viability after transfection with PDMAEMA, PMETAC and CS-PMETAC brush coated nanoparticles (forming siRNA complexes at N/P ratios of 10 and 20), compared with lipofectamine (results presented in Supplementary Figure 15; n.s.: not significant, *: $p<0.05$, **: $p<0.01$, , ***: $p<0.001$).

Groups compared with lipofectamine	Day 1	Day 2	Day 3	Day 4
	Significance (*p* value)			
SiO_2-PDMAEMA10	0.003406	0.158441	0.498621	0.000251
SiO_2-PDMAEMA20	0.111750	0.017451	0.000559	0.006519
SiO_2-PMETAC10	0.000003	0.027370	0.031637	0.242214
SiO_2-PMETAC20	0.000020	0.042614	0.012320	0.472217
SiO_2-CS-PMETAC10	0.000020	0.029468	0.047266	0.553964
SiO_2-CS-PMETAC20	0.000014	0.027929	0.009731	0.940702

Supplementary Table 6. Summary of statistical analysis for HaCaT-GFP transfections with PDMAEMA, PMETAC and CS-PMETAC brush coated nanoparticles (forming siRNA complexes at N/P ratios of 10 and 20), compared with lipofectamine (results presented in Figure 6G; n.s.: not significant, *: $p<0.05$, **: $p<0.01$).

Groups compared with lipofectamine	Day 1	Day 2	Day 3	Day 4
	Significance (*p* value)			
SiO_2-PDMAEMA10	0.0363	0.1220	0.1480	0.0046
SiO_2-PDMAEMA20	0.3952	0.6101	0.1916	0.0193
SiO_2-PMETAC10	0.0051	0.0352	0.0952	0.0051
SiO_2-PMETAC20	0.0527	0.0001	0.0137	0.0012
SiO_2-CS-PMETAC10	0.0016	0.0005	0.0003	0.0854
SiO_2-CS-PMETAC20	0.0018	0.0015	0.0025	0.6818

Significance comparison of transfection between different days for each reagent (*p* value)						
	D1 vs D2	D1 vs D3	D1 vs D4	D2 vs D3	D2 vs D4	D3 vs D4
Lipo	0.9690	0.8005	0.0115	0.7602	0.0069	0.0097
SiO_2-PDMAEMA10	0.8218	0.0136	0.0052	0.0401	0.0441	0.5294
SiO_2-PDMAEMA20	0.8708	0.1469	0.3783	0.3144	0.5849	0.3616
SiO_2-PMETAC10	0.0025	0.0024	0.0029	0.5234	0.1732	0.3000
SiO_2-PMETAC20	0.0046	0.0106	0.0067	0.0038	0.4788	0.0783
SiO_2-CS-PMETAC10	0.3093	0.2147	0.7422	0.8738	0.7212	0.6446
SiO_2-CS-PMETAC20	0.4739	0.0721	0.1469	0.1910	0.3106	0.9635

Supplementary Table 7. Statistical analysis for HaCaT-GFP transfection on day 10 with PMETAC brush coated nanoparticles forming complexes with siRNA at a N/P ratio of 10, compared with lipofectamine (results presented in Figure 6H; *: $p<0.05$).

Group compared with lipofectamine	Day 10
	Significance (*p* value)
SiO_2-PMETAC10	0.0373

References

1 Eldridge, A. G., Li, Y., Sharp, P. A. & Blencowe, B. J. The SRm160y300 splicing coactivator is required for exon-enhancer function. *Proc. Natl. Acad. Sci.* **96**, 6125-6130 (1999).

2 Rai, A. *et al.* bMERB domains are bivalent Rab8 family effectors evolved by gene duplication. *eLife* **5**, e18675 (2016).

3 Harbour, M. E., Breusegem, S. Y. & Seaman, M. N. J. Recruitment of the endosomal WASH complex is mediated by the extended 'tail' of Fam21 binding to the retromer protein Vps35. *Biochem. J* **442**, 209-220 (2012).

4 Demaegd, D. *et al.* Newly characterized Golgi-localized family of proteins is involved in calcium and pH homeostasis in yeast and human cells. *Proc. Natl. Acad. Sci.* **110**, 6859-6864 (2013).

5 Qu, F., Li, D., Ma, X., Chen, F. & Gautrot, J. E. A kinetic model of oligonucleotide-brush interactions for the rational design of gene delivery vectors. *Biomacromolecules* **20**, 2218-2229 (2019).

6 Krishnamoorthy, M. *et al.* Solution conformation of polymer brushes determines their interactions with DNA and transfection efficiency. *Biomacromolecules* **18**, 4121-4132 (2017).

7 Koopal, L. K. & Avena, M. J. A simple model for adsorption kinetics at charged solid-liquid interfaces. *Colloids Surf. A* **192**, 93-107 (2001).
