# Supplementary material for: Competitive binding and molecular crowding regulate the cytoplasmic interactome of non-viral polymeric gene delivery vectors
Source: Nat Commun. 2021 Nov 8;12:6445. doi: 10.1038/s41467-021-26695-w (PMC8576037; doi:10.1038/s41467-021-26695-w)
Supplement: Supplementary file 3 — Reporting summary [file 41467_2021_26695_MOESM3_ESM.pdf]

nature research

Corresponding author(s): Julien Gautrot

Last updated by author(s): Sep 16, 2021

Reporting Summary

Nature Research wishes to improve the reproducibility of the work that we publish. This form provides structure for consistency and transparency in reporting. For further information on Nature Research policies, see our Editorial Policies and the Editorial Policy Checklist.

Statistics

For all statistical analyses, confirm that the following items are present in the figure legend, table legend, main text, or Methods section.

n/a	Confirmed	
☐	☒	The exact sample size (n) for each experimental group/condition, given as a discrete number and unit of measurement
☐	☒	A statement on whether measurements were taken from distinct samples or whether the same sample was measured repeatedly
☐	☒	The statistical test(s) used AND whether they are one- or two-sided<br>*Only common tests should be described solely by name; describe more complex techniques in the Methods section.*
☐	☒	A description of all covariates tested
☐	☒	A description of any assumptions or corrections, such as tests of normality and adjustment for multiple comparisons
☐	☒	A full description of the statistical parameters including central tendency (e.g. means) or other basic estimates (e.g. regression coefficient) AND variation (e.g. standard deviation) or associated estimates of uncertainty (e.g. confidence intervals)
☐	☒	For null hypothesis testing, the test statistic (e.g. F, t, r) with confidence intervals, effect sizes, degrees of freedom and P value noted<br>*Give P values as exact values whenever suitable.*
☒	☐	For Bayesian analysis, information on the choice of priors and Markov chain Monte Carlo settings
☒	☐	For hierarchical and complex designs, identification of the appropriate level for tests and full reporting of outcomes
☒	☐	Estimates of effect sizes (e.g. Cohen's d, Pearson's r), indicating how they were calculated

Our web collection on statistics for biologists contains articles on many of the points above.

Software and code

Policy information about availability of computer code

Data collection	Mass spectrometry data was recorded using Xcalibur software (version 4.0). DLS/zetasizing data was acquired using 7.02 Malvern Zetasizer Software. FTIR data was recorded on OPUS 8.5.29. Ellipsometry data was acquired using CompleteEASE. Photographs of gels after electrophoresis were taken using a Molecular Imager system (Gel Doc XR, BIO RAD). NMR spectra were recorded using Bruker IconNMR 5.0.9 Build 47 (For TopSpin 3.6.1). TGA data was recorded on TA Q Series Version 2.5.0.256. SPR data was recorded using the Biacore X Control Software. Epifluorescence images were acquired on a Leica DMI8, with the Leica Application Suite X (3.4.2.18368). Confocal microscopy was carried out on a Zeiss lsm 710 using Zen 2012 sp5.
Data analysis	No custom-made software was used for data analysis. Quantitative analysis of microscopy images was carried out with ImageJ. Statistical analyses were performed using Origin 8.

For manuscripts utilizing custom algorithms or software that are central to the research but not yet described in published literature, software must be made available to editors and reviewers. We strongly encourage code deposition in a community repository (e.g. GitHub). See the Nature Research guidelines for submitting code & software for further information.

Data

Policy information about availability of data

All manuscripts must include a data availability statement. This statement should provide the following information, where applicable:

- Accession codes, unique identifiers, or web links for publicly available datasets
- A list of figures that have associated raw data
- A description of any restrictions on data availability

The data that support the findings of this study are available from the corresponding author upon reasonable request. Source datasets are also available from the Nat. Comm. website, and have been deposited into a QMUL repository. Proteomics data generated in this study have been deposited in the PRIDE database under

accession code PXD028512 [Project DOI: 10.6019/PXD028512].Full details of the proteomics data will be made available freely.

Field-specific reporting

Please select the one below that is the best fit for your research. If you are not sure, read the appropriate sections before making your selection.

☒ Life sciences ☐ Behavioural & social sciences ☐ Ecological, evolutionary & environmental sciences

For a reference copy of the document with all sections, see nature.com/documents/nr-reporting-summary-flat.pdf

Life sciences study design

All studies must disclose on these points even when the disclosure is negative.

Sample size	Proteomics data was collected from two separate experiments. All other experiments were carried out in triplicate. No statistical method was used to predetermine the sample size. Sample sizes in this study were estimated based on common practice in the field.
Data exclusions	No data was excluded.
Replication	Replication of data was carried out in independent separate experiments (in triplicate). No data was discarded.
Randomization	In line with common practice in the field, experiments were not randomized.
Blinding	In line with common practice in the field, blinding was not used for data analysis and quantification.

Reporting for specific materials, systems and methods

We require information from authors about some types of materials, experimental systems and methods used in many studies. Here, indicate whether each material, system or method listed is relevant to your study. If you are not sure if a list item applies to your research, read the appropriate section before selecting a response.

Materials & experimental systems

n/a	Involved in the study	
☒	☐	Antibodies
☐	☒	Eukaryotic cell lines
☒	☐	Palaeontology and archaeology
☒	☐	Animals and other organisms
☒	☐	Human research participants
☒	☐	Clinical data
☒	☐	Dual use research of concern

Methods

n/a	Involved in the study	
☒	☐	ChIP-seq
☒	☐	Flow cytometry
☒	☐	MRI-based neuroimaging

Eukaryotic cell lines

Policy information about cell lines

Cell line source(s)	HCA2 cells were obtained from Dr Mat Caley (QMUL; original description can be found in HCA2: J Cell Sci 2004;117:3389-403). HaCaT cells were obtained from Prof. David Kelsell (QMUL; original description can be found in J. Cell Biol. 106 (1988) 761-771)
Authentication	Cell lines were not further authenticated (HCA2: J Cell Sci 2004;117:3389-403; HaCaTs: J. Cell Biol. 106 (1988) 761-771). Cell morphologies are consistent with initial reports and, in the case of HaCaT cells, expression of epidermal keratins was confirmed.
Mycoplasma contamination	Cell lines are routinely tested negatively for mycoplasma
Commonly misidentified lines (See ICLAC register)	No commonly misidentified cell lines were used in this study.
