# Supplementary material for: Competitive binding and molecular crowding regulate the cytoplasmic interactome of non-viral polymeric gene delivery vectors
Source: Nat Commun. 2021 Nov 8;12:6445. doi: 10.1038/s41467-021-26695-w (PMC8576037; doi:10.1038/s41467-021-26695-w)

L - Ladder (molecular distribution ranging from 100 to 10000 bp)
Si - Bare siRNA (22 bp)
1,2 - PDMAEMA brush nanoparticles incubated in cell lysate and treated with 2 M NaCl
3,4 - PMETAC brush nanoparticles incubated in cell lysate and treated with 2 M NaCl

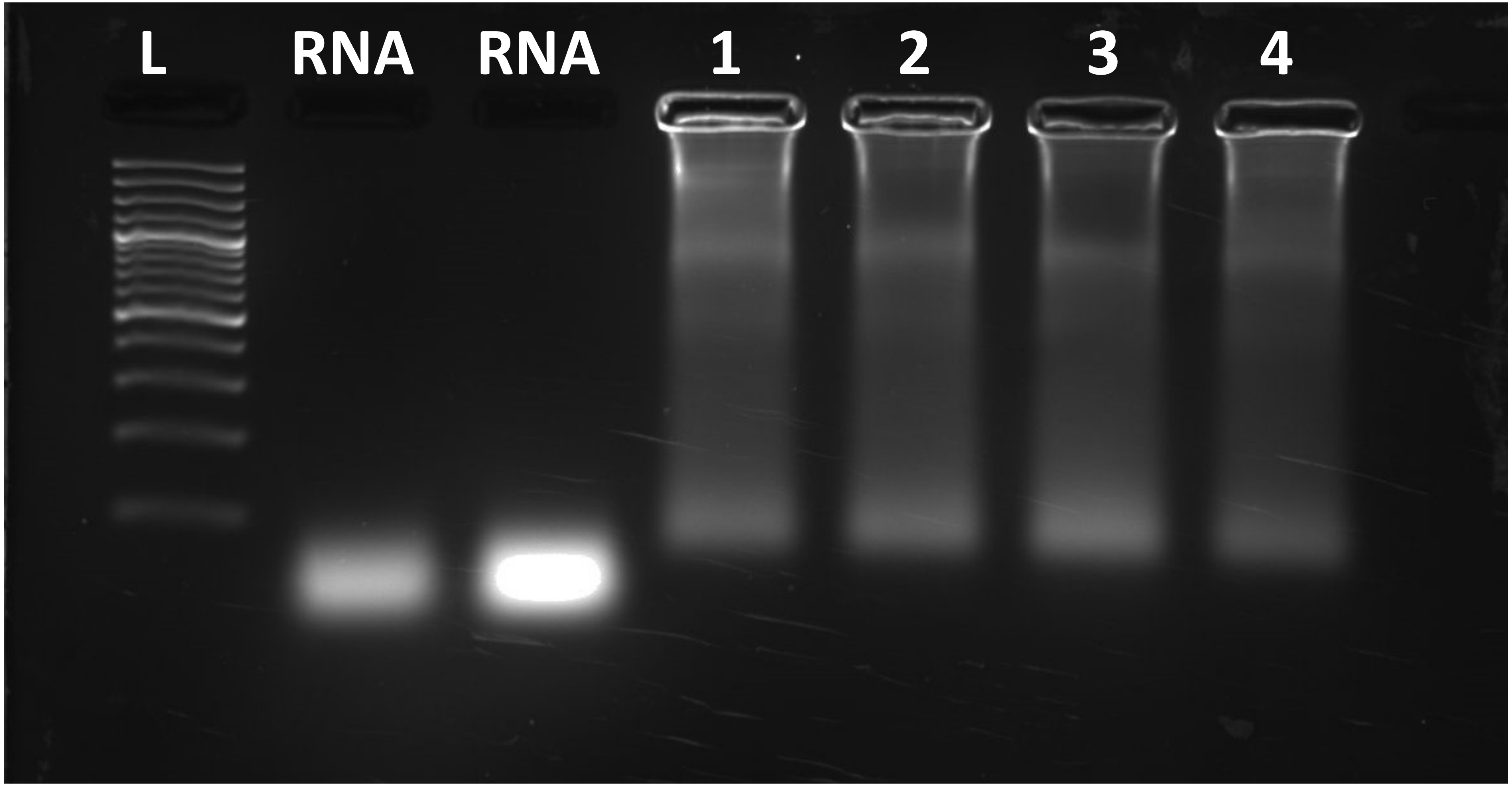

Supplement: Supplementary file 4 — Source data [file 41467_2021_26695_MOESM4_ESM.zip › Source Data/Uncropped Gel - Figure 1D.pdf]
